# Real-time estimation of the epidemic reproduction number: Scoping review of the applications and challenges

**DOI:** 10.1371/journal.pdig.0000052

**Published:** 2022-06-27

**Authors:** Rebecca K. Nash, Pierre Nouvellet, Anne Cori

**Affiliations:** 1 MRC Centre for Global Infectious Disease Analysis, Jameel Institute, School of Public Health, Imperial College London; 2 School of Life Sciences, University of Sussex; ISI Foundation: Fondazione ISI - Istituto per l’lnterscambio Scientifico, ITALY

## Abstract

The time-varying reproduction number (R_t_) is an important measure of transmissibility during outbreaks. Estimating whether and how rapidly an outbreak is growing (R_t_ > 1) or declining (R_t_ < 1) can inform the design, monitoring and adjustment of control measures in real-time. We use a popular R package for R_t_ estimation, EpiEstim, as a case study to evaluate the contexts in which R_t_ estimation methods have been used and identify unmet needs which would enable broader applicability of these methods in real-time. A scoping review, complemented by a small EpiEstim user survey, highlight issues with the current approaches, including the quality of input incidence data, the inability to account for geographical factors, and other methodological issues. We summarise the methods and software developed to tackle the problems identified, but conclude that significant gaps remain which should be addressed to enable easier, more robust and applicable estimation of R_t_ during epidemics.

## Author summary

Many software tools have been developed to support real-time outbreak response, allowing epidemiologists and other health professionals to characterise new and persistent pathogens and their threat to populations. Some of these tools allow the user to estimate how rapidly a pathogen is spreading through a population in real-time. This knowledge is crucial for a timely and effective response, helping to inform the optimal choice of control strategies. However, these tools are not perfect, and may need to be adapted to make them applicable to more pathogens and a variety of contexts. Our study summarises the different tools available in the literature and identifies the remaining issues that are yet to be addressed. We combine these findings with feedback from users of a popular tool, EpiEstim, to better understand the key priorities for development going forward.

## Introduction

Transmissibility quantifies how easily a pathogen can spread through a population and can depend on numerous factors, such as the pathogenicity of the infectious agent but also the current level of immunity, demographics, and connectivity in the population. Transmissibility is typically measured by the time-varying (or effective) reproduction number (R_t_), which represents the average number of secondary infections generated by a case at time t of an outbreak.[1–4] R_t_ is an important element of outbreak analysis as it indicates whether case numbers are rising (R_t_ > 1) or falling (R_t_ < 1) and by how much. This can guide intervention planning and help to determine whether current control measures are effective, and if not, to what extent they need to be intensified. R_t_ estimates can also feed into real-time incidence forecasts, which can assist logistics and resource planning, for instance, by identifying whether hospital bed capacity is likely to be exceeded or if resources need to be allocated to specific areas.[5–8]

Many methods have been developed to estimate R_t_ in real-time; one of the most commonly used is through the renewal equation (Eq 1), which relies on a branching process model. The model assumes that the incidence of new cases on day t (I_t_) can be represented by a Poisson process:

$$I_{t}∼Pois(R_{t}\sum_{s=1}^{t}I_{t-s}\omega_{s})$$ (1)

where R_t_ is the time-varying reproduction number (i.e. the average number of cases caused by a primary case infected at time t, assuming that conditions remain the same after time t), and the past incidence (I_t-s_) is weighted by ω_s_, the probability mass function of the generation time (the time between infection in a case and their infector).[3, 9] In practice, as infection itself is difficult to observe, the incidence of symptomatic cases can be used instead and ω_s_ can be approximated by the serial interval (SI, the time between symptom onset in a case and their infector. See Britton and Scalia Tomba for the implications of this approximation).[10] Cori et al. developed a method to estimate R_t_ from the renewal equation that is suitable for real-time application.[11] The method is implemented in the R package EpiEstim, where estimation is performed over user-defined time windows within which R_t_ is assumed constant. Longer windows typically lead to smoother estimates, but may artificially hide some of the temporal variability.[12]

**Fig 1:**
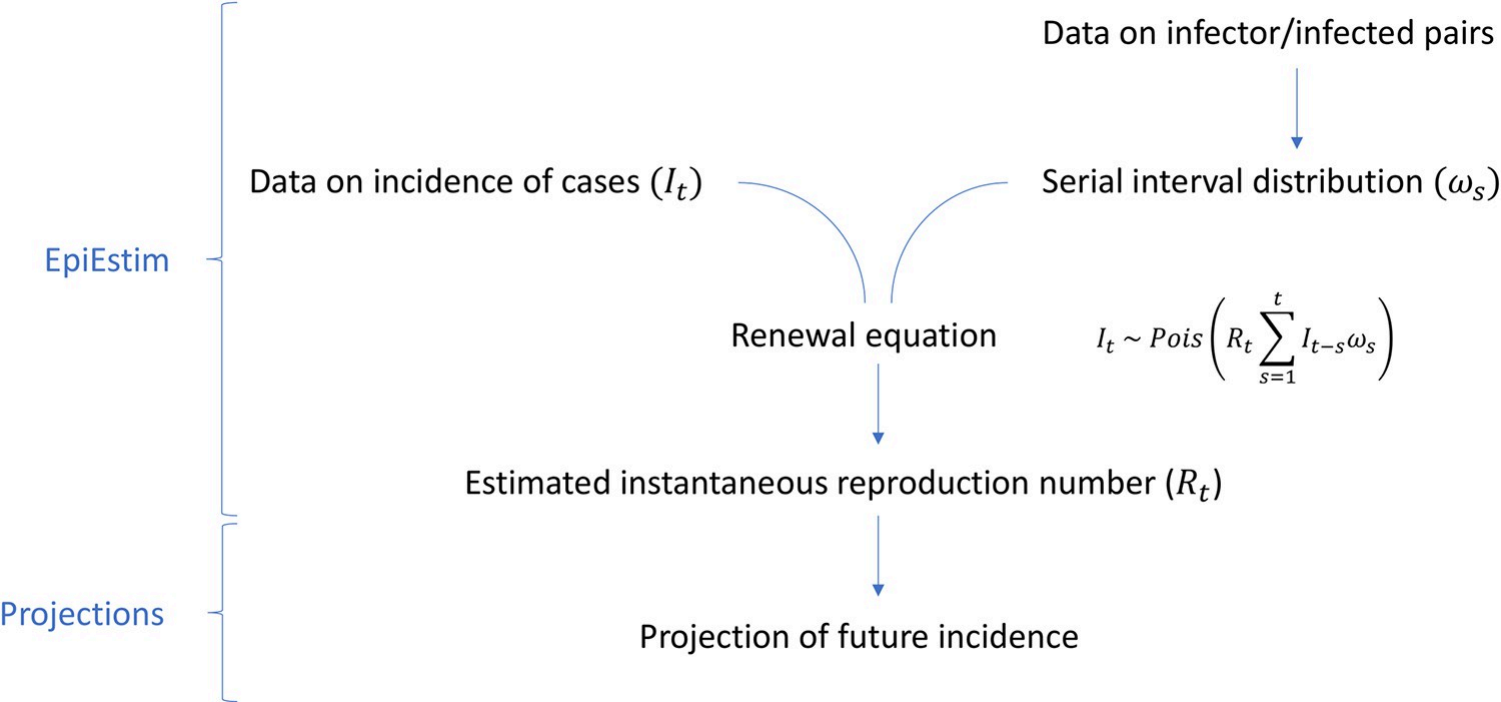
Schematic of the forecasting process. In the renewal equation, the incidence at time t (I_t_) is expressed as a function of the serial interval distribution (ω_s_), the time-varying reproduction number (R_t_) and the past incidence (I_t-s_).

R_t_ estimates can then be used to project forward in time and forecast future epidemic trajectories (using Eq 1, see Fig 1), as implemented in the R package projections.[13]

The COVID-19 pandemic has generated a surge in methodological and tool developments to estimate R_t_, as well as numerous applications of these methods. We aimed to describe the landscape of methods and real-world applications of R_t_ estimation, and to identify potential gaps that could be addressed to make those methods more easily applicable and more useful in practice in the future.

EpiEstim has been widely used for R_t_ estimation during the COVID-19 pandemic and has been shown to perform better than many other methods in terms of estimation accuracy.[12] Therefore, we use EpiEstim as a starting point to identify ways in which renewal equation methods have been used and modified.[12] We reviewed research articles that either used EpiEstim (the method or the software) or described a modified approach to address unmet needs. Alongside this scoping literature review, a questionnaire was distributed to known users of EpiEstim and advertised on social media to gather information on issues that were unlikely to be described in publications, e.g. computational speed or usability. By collating the findings, we aim to reveal some of the key challenges when estimating R_t_ in real-time and those that are yet to be addressed.

## Methods

### Scoping review: Search strategy and selection criteria

Google scholar was used to identify all articles or reports up to 10^th^ December 2020 that cited one of the two papers describing EpiEstim (Cori et al. 2013 or Thompson et al. 2019).[3, 14] After full text screening, two databases were compiled using the inclusion and exclusion criteria outlined in Table 1, collating papers or reports that used: i) an unmodified version of the EpiEstim method or software and ii) a modified version of the EpiEstim method or software.

**Table 1:** Inclusion and exclusion criteria for the scoping review.

Inclusion for unmodified papers	Inclusion for modified papers	Exclusion
◾ Cited Cori 2013 or Thompson 2019. ◾ Did not mention any alterations to the original method.	◾ Cited Cori 2013 or Thompson 2019. ◾ Described altering the approach e.g. used a method that is “similar to…” or “modified from…” EpiEstim.	◾ Did not implement the method or a modified method. ◾ Pre-print that has been updated (by 31^st^ March 2021) and no longer cites the paper(s). ◾ Duplicate (e.g. updated pre-print versions with different names). ◾ Withdrawn pre-print.

### Evaluation of R package or tool usability

Each R package or tool identified in the scoping review was appraised in terms of its ease of installation, available documentation (e.g. vignette or tutorials) and speed of estimation of the reproduction number (see Table B and section 3 in S1 Text for more detail).

### Questionnaire

We designed an online questionnaire including 16 questions aiming to reveal the key challenges users encountered when using EpiEstim to estimate R_t_. It was shared via Twitter and distributed to a list of 41 known EpiEstim users.[15] The questionnaire was live between 11^th^ and 31^st^ January 2021.

### Ethics statement

Ethical approval was not required as the questionnaire was evaluative and solely to understand the current use and limitations of the EpiEstim R package. Respondents were informed that their survey responses were being collected for academic purposes.

## Results

**Fig 2:**
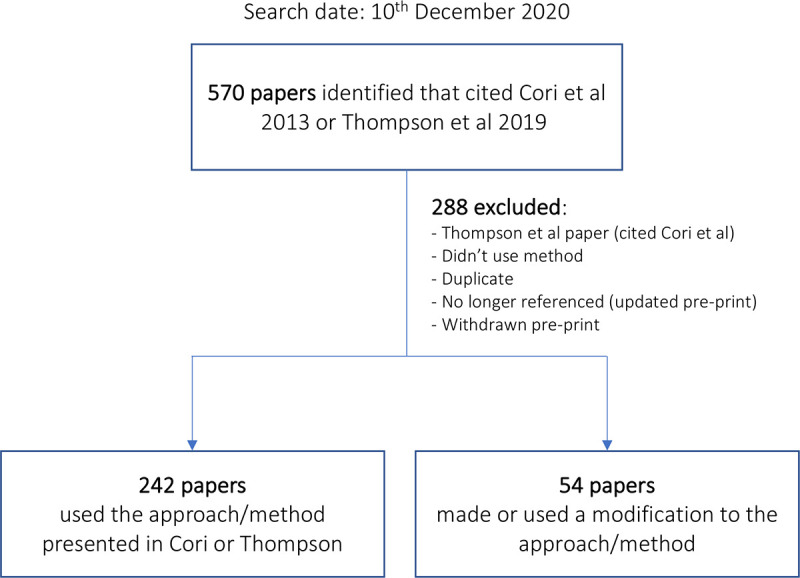
Schematic to illustrate the scoping review process. Note: fifteen papers are counted as both unmodified and modified (i.e., EpiEstim was used as a baseline to compare a modified approach to).

281 papers were included, which cited the original Cori et al. 2013 paper or the paper by Thompson et al. 2019 describing the latest version of EpiEstim (Fig 2).[3, 14] Papers investigated a variety of diseases; the most common was COVID-19, followed by Ebola and influenza (Figure A in S1 Text). The majority (*n* = 242) had used an unmodified version of EpiEstim (Table A in S1 Text), and 54 papers described at least one modification.[7, 16–67] Fifteen papers fell into both categories i.e., used the original EpiEstim method as a baseline to compare a new approach to.[16–30]

**Fig 3:**
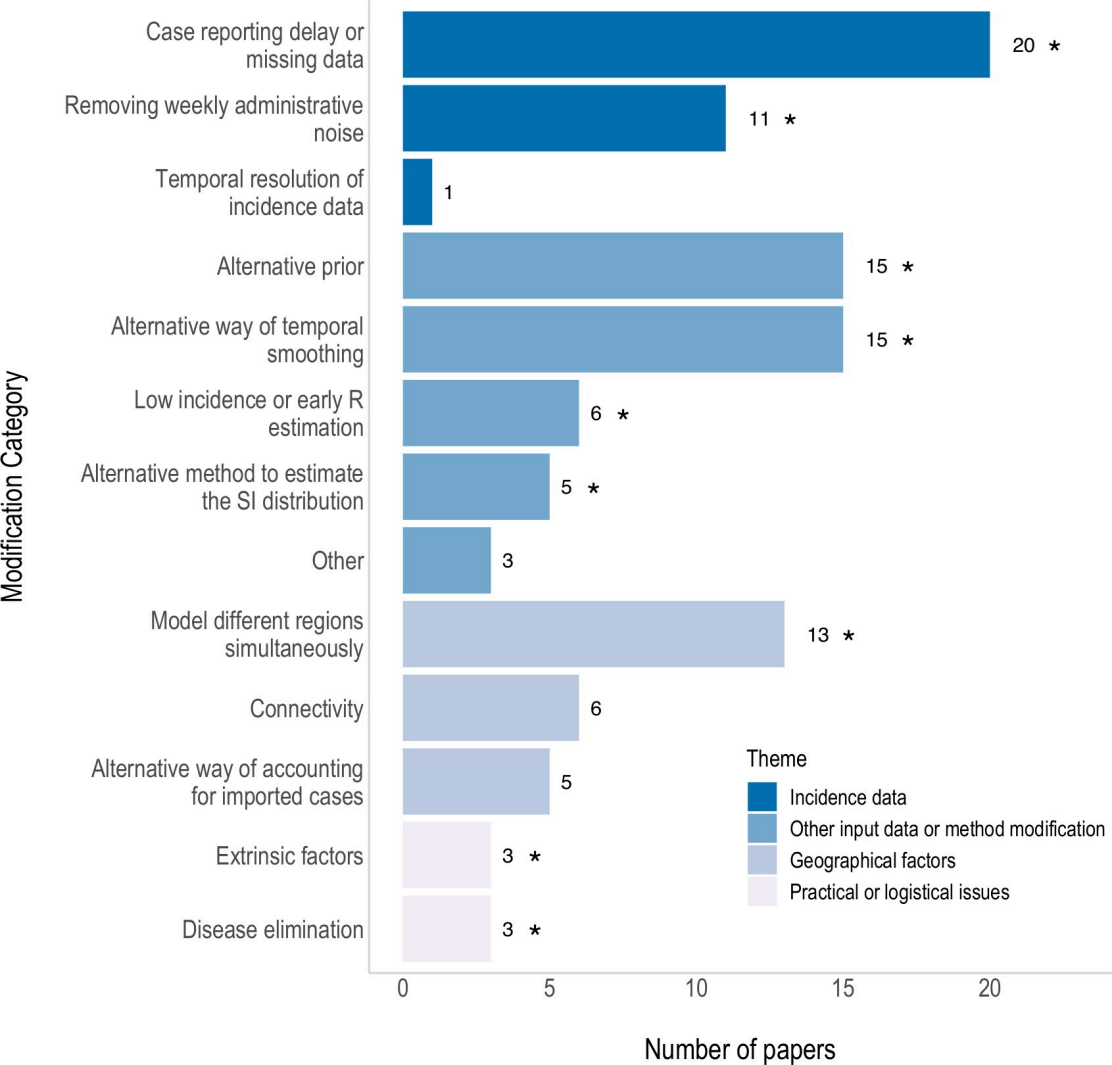
Summary of the modifications made to the EpiEstim method in 54 papers. Papers which made multiple modifications are counted more than once. Asterisks are present next to the modification category if an R package or opensource tool has been identified that addresses an issue within that category. See text for a description of all modifications and Table 2 for a summary of the R packages identified.

We classified the 106 issues addressed in the 54 papers proposing modifications into 13 categories (Fig 3). The most common included delays in the reporting of cases or missing data (*n* = 20), weekly administrative noise (*n* = 11), choice of prior for R_t_ (*n* = 15), and modelling different regions at the same time (*n* = 13). Each modification type was further categorised into four themes: 1) incidence data, 2) other input data or method modification 3) extension to account for geographical or spatial factors, and 4) practical or logistical issues.

**Table 2:** Summary table of the R packages and tool (*) that address some of the issues identified in the scoping review. The ticks (✓) indicate whether an R package includes that modification type and rows that are grey show the modification types that have not been incorporated into a known R package or tool. The final three rows summarise additional exploration by the authors to assess how easily each package/tool can be installed and used. For full details of how each classification (very good = ✓✓, good = ✓, poor = ✗) was defined, see Table B in S1 Text and the associated text (section 3).

**Theme**	**Modification type**	**APEestim** *(v 0*.*0*.*1)*	**bayEStim** *(v 0*.*0*.*1)*	**earlyR**>break/> *(v 0*.*0*.*5)*	**epicontacts** *(v 1*.*1*.*2)*	**Epidemia** *(v 1*.*0*.*0)*	EpiFilter* (*Tool)	**EpiNow2** *(v 1*.*3*.*2)*
**Incidence**	Case reporting delay or missing data		✓			✓		✓
	Removing weekly administrative noise							✓
	Temporal resolution of incidence data							
**Other input data or method modification**	Low incidence or early R estimation			✓			✓	
	Alternative prior					✓	✓	✓
	Alternative way of temporal smoothing	✓				✓	✓	✓
	Alternative method to estimate the SI distribution				✓			
**Geographical factors**	Connectivity							
	Model different regions simultaneously					✓		✓
	Alternative way of accounting for imported cases							
**Practical/logistical**	Extrinsic factors					✓		
	Disease elimination						✓	
**Additional exploration** (✓✓ = very good, ✓ = good, ✗ = poor)								
**Usability**	Ease of installation	✓	✗	✓✓	✓✓	✗	✗	✓
	Documentation and tutorials (e.g., vignette)	✗	✗	✓✓	✓✓	✓✓	✓✓	✓✓
	Speed of R estimation^♢^ (^♢^except for epicontacts, which estimates the SI)	✓✓	NA	✓✓	✓✓^♢^	✓	✓✓	✗

### Modifications involving incidence data

The accuracy of R_t_ estimates directly depend on the quality of the input data, particularly the incidence data, which can be influenced by several factors. Incidence by date of infection is best suited to measure real-time transmission, but it is rarely available. A common workaround is to use incidence by date of symptom onset, resulting in “delayed” R_t_ estimates, representing transmission one incubation period before time t.[3] There are often additional delays between symptom onset and subsequent reporting of cases, leading to right censoring when observing cases in real time.[3] Recent incidence observed in real-time is therefore often underestimated, which can bias R_t_ estimates if not accounted for. This is further complicated when reporting delays vary over time, e.g. at the start of an outbreak when robust reporting systems are not yet in place. Beyond issues related to delays in reporting, some cases may not be reported at all; however this should not bias R_t_ estimates, unless reporting varies over short time scales e.g. if testing capacity fluctuates.[3]

Thirty percent of modifications (*n* = 32) addressed issues with incidence data. The most common was to account for reporting delays or missing data (i.e. underreporting or cases with missing date information) (*n* = 20).

The R packages bayEStim, EpiNow, and EpiNow2 (which is also used to generate R_t_ estimates for routine forecasting on the website EpiForecasts [52, 54]) all allow back-calculation of the incidence of symptom onset or infection from the incidence of reported cases using a reporting delay or incubation period distribution.[18, 25–27, 31, 47, 52, 54, 55, 68, 69] Such delays can be informed by individual case timelines or by characterising the delay after which reported incidence stabilises. Huisman et al. deconvolve over the observed incidence time series with a delay distribution that is specific to the type of case detection (e.g. positive test, hospital admission, death).[20] Li et al. use region and time dependent delay distributions.[23]

Some papers adjusted their approach to explicitly account for underreporting.[20, 23, 30, 41, 42] The R package Epidemia does so through a time-varying reporting rate.[37, 46, 70] A commonly adopted approach to circumvent time-varying case reporting is to use the incidence of deaths, as reporting of deaths is usually less prone to time fluctuations than reporting of cases. Nouvellet et al. and Djafaara et al. used COVID-19 death data to estimate R_t_ directly using the renewal equation.[17, 59] Others have estimated R_t_ from the incidence of infection or symptom onset, back-calculated from the observed incidence of death.[22, 44] Epidemia and EpiNow2 use a similar approach, and estimates can be based on incidence by date of infection, hospital admission or death.[18, 25–27, 31, 37, 46, 47, 52, 54]

The second most common modification was adjusting for weekly administrative noise (*n* = 11), where reporting can vary depending on the day of the week e.g. due to fewer tests being performed or longer processing times on the weekends.[71] As the default, EpiEstim estimates R_t_ over weekly time windows to smooth out these temporal trends. Modifications (including in EpiNow2) have been made to account for weekly noise in the incidence data by explicitly including a day of the week effect.[18, 25–27, 31, 34, 35, 47, 52, 54, 56]

One paper adjusted the temporal resolution of incidence data. Currently, EpiEstim requires the incidence data and the SI distribution to be on the same time scale. For acute infections, where the SI is short, this means weekly incidence is not supported and daily incidence is required as an input. Yamauchi et al. use EpiEstim on daily incidence inferred from weekly observations using smoothing splines.[66]

### Other input data or method modification

As the default, EpiEstim uses a gamma distributed prior for R_t_, with a mean and standard deviation of 5. Such a high mean value avoids estimating a subcritical reproduction number (R_t_ < 1) unless the data supports it, but can lead to overestimation of R_t_ in periods with low incidence. Priors for R_t_ are fixed across all time windows, but the length of the time window, which can be varied, determines the smoothness of the resulting estimates. Many methods have addressed the problem of temporal smoothness using alternative, often autoregressive, prior specifications.[18, 24–28, 30, 31, 37, 46, 47, 52, 54, 63, 64] Turbé et al. updated the gamma distributed prior for each time window with the previous posterior, so that estimates benefit from the information that becomes available over time.[28] Other methods, including those implemented in Epidemia, EpiNow2, and another opensource tool, EpiFilter, can control the temporal variation in R_t_ using random walks, such as gaussian processes.[18, 24–27, 30, 31, 37, 46, 47, 52, 54, 63, 64, 72] In Epidemia, in addition to being smoothed using a random walk, R_t_ can also be constrained by covariates such as mobility, and these covariates themselves are given priors.[37, 46]

Overall, fifteen papers described modifications related to the temporal smoothing of successive R_t_ estimates, which in EpiEstim involves a subjective time window choice over which each R_t_ is estimated. Choosing a time window length is a trade-off between identifying important temporal trends and smoothing out random noise in the data. Pathogens with short SIs and/or in the context of fast-changing transmission (e.g. due to control measures) will require shorter time windows to capture the former, while in low incidence settings, longer time windows may be needed to encompass more data. Four papers use metrics (either the Accumulated Prediction Error, implemented in the R package called APEestim, or the Fisher Information Approximation) to identify the optimal time window.[19, 24, 60, 61, 73] Instead of relying on a time window, methods which can control temporal variation using random walks can incorporate parameters which control the autocorrelation of successive R_t_ estimates.[18, 24–27, 30, 31, 47, 52, 54, 63, 64]

Six papers addressed issues with R_t_ estimation when incidence is low, where EpiEstim would typically return the prior.[21, 24, 43, 49, 50, 63] The R package earlyR is the frequentist equivalent of EpiEstim, and therefore returns estimates that are only data-driven, but it is not set up to estimate time-varying reproduction numbers.[21, 43, 49, 50, 74] EpiFilter can use data before and beyond time t to estimate R_t_, which means that in addition to real-time estimation it can facilitate more robust retrospective estimation, particularly in low incidence settings.[24, 63]

In EpiEstim, the SI distribution can be specified as parametric (where only the mean and standard deviation are required), non-parametric (where the whole discrete distribution is provided), or “uncertain”, where a range of potential SIs are considered. In 2019, Thompson et al. extended the framework so that the SI distribution can be directly estimated within EpiEstim from data on known pairs of primary and secondary cases.[14] Five papers propose alternative approaches to derive the SI that they subsequently use in EpiEstim or another R package.[33, 43, 45, 51, 67] Al-Wahaibi et al. used an alternative R package, Epicontacts, to estimate the SI distribution directly from contact tracing data.[33, 75] Donnat and Holmes effectively assume a linearly decreasing probability mass function for the SI with parameters optimised based on past incidence data.[43] Knight et al. explored a method to permit distributions that allow negative SI values, which occur when transmission can take place before symptom onset as e.g. for COVID-19.[51] For applicability to vector-borne disease, some approaches modelled the SI as a distribution for both the human and vector.[45, 67]

Less common methodological modifications included aiming to improve the quantification of uncertainty using a Bayesian filtering method [39] or jointly estimating the reproduction number and the early incidence to account for highly variable reporting over time.[4, 7]

### Modifications involving geographical factors

The simple renewal equation detailed in Eq 1 describes transmission in a single closed location. Thirteen papers developed or used methods that allow R_t_ to be estimated simultaneously for different locations. EpiNow2 simply adds the functionality to supply incidence for multiple locations at once and runs the same model to estimate R_t_ for each region in turn, producing a joint output facilitating comparisons between locations.[18, 25–27, 31, 47, 52, 54] Abry et al.’s approach enables simultaneous estimation of R_t_ from incidence time series in different locations, either independently or jointly using spatial smoothing.[32] Some approaches model transmission in different regions using linear predictors (which can include environmental, demographic, or intervention related factors), as in Epidemia, where parameters can be fixed or varying by location.[37, 46, 56, 64]

Six papers further modified the approach to explicitly account for connectivity.[36, 40, 53, 58, 59, 66] To account for connectivity between individuals or locations, Ng et al. weighted the probability of transmission between two cases depending on the distance between them, [58] while other studies inferred connectivity between locations using a gravity model approach.[36, 40] Chirombo et al. developed an estimator for a spatiotemporal R that accounts for susceptibility and infectiousness levels in each location and connectivity between them.[40] Backer et al. identified areas with a key role in disease spread by combining estimates of location-specific R_t_ with estimated movements out of each location to other areas.[36]

Transmissibility may also be constrained by explicitly using mobility data.[53, 59, 66] Yamauchi et al. used a similar approach to Backer et al. but used census data to inform movement between jurisdictions.[36, 66] In a less specific way, several studies examined the extent to which reduction in the level of mobility due to social distancing led to reductions in R_t_.[53, 59]

A simpler approach to estimate R_t_ for a location whilst accounting for cases arising elsewhere is to distinguish between imported and locally acquired cases, as proposed in the EpiEstim extension by Thompson et al.[14] Five other papers described similar modifications to account for imported cases or for an exogenous force of infection.[38, 48, 57, 63, 65] Bourhy et al. extended the renewal equation to incorporate the rate of introduction of rabies into a city.[38] Two papers estimated R_t_ separately for locally acquired versus overseas acquired cases in order to model the effects of different interventions targeted at each group.[48, 65]

### Modifications with practical or logistical applications

R_t_ estimation is often used as the basis for practical decision making in response to changing transmissibility in real-time. For example, the implementation of interventions, such as social distancing measures, estimating the adequacy of current control measures, or deciding when to declare that an outbreak has ended.[16, 76–78] Despite the clear link between R_t_ estimation and policymaking, very few modifications (let alone R packages) have been made to directly answer important practical or logistical questions.

Modifications were made to estimate the impact of potential drivers of transmission on R_t_ estimates (*n* = 3). Wang et al. modelled the effects of interventions by incorporating additional parameters to explicitly account for the impact of interventions on the infectious period or the overall level of infectiousness of infected cases.[29] Epidemia accounts for extrinsic factors, such as interventions and changes in mobility, by creating a linear predictor which includes the factors influencing changes in transmission.[37, 46]

Three papers sought to develop probabilistic methods to predict local disease elimination or the risk of epidemic resurgence, one of which is implemented in EpiFilter.[16, 62, 63]

A summary of the R packages and software tool that incorporate some of the modifications identified through the literature review is available in Table 2. We also performed additional exploration to assess their usability and the speed of estimation of the reproduction number (when relevant examples were provided). This is a non-comprehensive assessment based on the R packages/tools available at the time of writing (see Table B and section 3 in S1 Text and the documentation of each package for more details). Two of the seven were very easy to install, and the majority (*n* = 5) provided documentation and vignettes. Of the R packages/tool that estimate the reproduction number, three were able to perform the estimation (using provided examples) in under a minute, however, only earlyR scored full marks in the usability category.

### Questionnaire

The questionnaire had 17 responses from 11 countries (Figure B in S1 Text). The majority of respondents were academic researchers, public health consultants or health professionals (Figure C in S1 Text). The small sample size makes it difficult to statistically analyse the responses, but overall, the questionnaire respondents echoed the broad themes identified during the scoping review (Fig 3, Table C in S1 Text). In addition, three other themes were identified: usability, speed and compatibility (Table C in S1 Text).

The most common issue affecting 53% of questionnaire respondents was usability, e.g. difficulties in using the package (at least initially–see p5 in S1 Text). Respondents suggested links to more resources, such as R code examples, to demonstrate how to apply and interpret outputs of EpiEstim in multiple scenarios. Speed issues were reported by 18% of questionnaire respondents, all using the ‘uncertain_si’ feature, where R_t_ estimates are integrated over a range of serial interval distributions. Compatibility issues were reported by users wanting to use alternative programming languages, such as JAGS or Julia, or wanting to use EpiEstim in a workflow with other R packages and suggested the addition of information on how to do so (see section 4 in S1 Text for further details).

## Discussion

To improve methods of R_t_ estimation, it is crucial to first gain an understanding of the current key challenges preventing, or restricting, practical applications of these methods to quantify pathogen transmissibility in real-time. To get an overview of such issues, we chose EpiEstim, one of the most popular and best-performing methods for estimating R_t_ in real-time, as a case study.[12] We analysed the literature citing EpiEstim as well as direct feedback from EpiEstim users collected through a questionnaire. We have also summarised the R packages identified that use alternative methodologies.

The most common challenge identified was to deal with factors influencing the quality of incidence data. Addressing frequently encountered issues, such as delays in reporting, underreporting, and administrative noise, appeared to be a priority in the field. Delays in reporting of cases or time-varying reporting rates have been a particular issue throughout the COVID-19 pandemic, linked to a high proportion of asymptomatic cases, fluctuations in testing, and health systems being under strain.[79–81] To counter some of these issues, R packages such as bayEStim and EpiNow2 explicitly account for delay distributions enabling “nowcasting”, which aims to eliminate any time-lag when estimating the impact of control measures or factors such as changes in behaviour.[68, 69]

When incidence by date of infection or symptom onset was unavailable or unreliable, some approaches used incidence by date of death. For instance, COVID-19 case data was initially very sporadic, whilst death data was more reliably reported. The R packages Epidemia and EpiNow2 allow the use of death data to maximise the use of available information in real-time.[69, 70] In many epidemics, including COVID-19, the reliability of case data improves over time and can provide more up-to-date insights into changing transmission dynamics. The ability to use either dataset means they can complement each other during different phases of the outbreak. However, the suitability of the type of data used will depend on the pathogen under investigation and the local context.

An issue that, to our knowledge, has yet to be addressed by any R package is the ability to estimate R_t_ using weekly-aggregated incidence data, which was highlighted in questionnaire responses and is a frequent query in correspondence with users of EpiEstim. This is a key ongoing issue, particularly for diseases such as influenza and Zika, where incidence is typically reported on a weekly basis, [45, 82] but also for COVID-19, with several US states having recently moved from reporting daily to weekly cases only.[83] While it is possible to supply EpiEstim and EpiNow2 with weekly-aggregated data if the SI is provided on the same time scale, aggregating a short SI to a weekly distribution may affect the quality of the R_t_ estimates and is not possible when the SI is shorter than a week.[45]

The scoping review identified additional methodological or data-related issues. These led to the development of methods that are more appropriate for use during periods of low incidence, [21, 24, 43, 49, 50, 63] or offer alternatives to allow less subjective and more flexible inputs into EpiEstim, e.g. the prior for R_t_, the SI distribution, or an alternative way of temporally smoothing R_t_ estimates.[18, 19, 21, 24–28, 30, 31, 33, 37, 43, 45–47, 49–52, 54, 60, 61, 63, 64, 67]

Modifications concerning geographical factors were also common in the literature, although only mentioned by one questionnaire respondent. Beyond accounting for imported cases [14, 63] and estimating R_t_ across different regions at the same time, [18, 25, 27, 31, 37, 46, 47, 52, 54] we found no R packages that explicitly addressed spatial interactions. This means that, aside from bespoke studies, [36, 40, 53, 56, 58, 59, 64, 66] important aspects of the spatial structure in transmission may have been overlooked. For instance, to our knowledge, there is no ready-to-use tool to analyse how easing restrictions in an area may influence transmission in neighbouring locations.

Another theme highlighted in both the scoping review and questionnaire responses was practical or logistical issues, such as evaluating or accounting for the impact of interventions, assessing when elimination has been reached, or extending the framework for logistical planning in hospitals. Transmissibility estimates ultimately inform intervention design, e.g. to select interventions that appear most effective and identify locations which should be targeted for implementation. The ability to estimate the impact of interventions on R_t_ across different countries is a useful feature of Epidemia, which uses a logistic regression framework to infer the impact of non-pharmaceutical interventions (e.g. social distancing) and reductions in mobility on transmission.[70] Additionally, an important practical consideration is when one can confidently estimate that elimination has been reached, which has been addressed by EpiFilter.[24, 63, 72] More R packages that directly address a variety of recurring logistical issues is a promising avenue for future research in the field.

Despite the small sample size (*n* = 17) and the imperfect nature of the questionnaire (see section 4 of S1 Text), it reinforced many of the findings from the scoping review and revealed additional challenges with computational speed, usability, and compatibility. We evaluated each of the identified R packages and tool based on these criteria and found that, of the packages that estimate the reproduction number, only earlyR scored full marks in the usability category (Table 2, Table B in S1 Text). These themes emphasise that package developers (of both new and existing methods) should ensure that increased functionality does not come at the expense of speed, and that additional resources are available to aid understanding of the package and how it can be used in conjunction with others in an outbreak analysis workflow. In response, we created a new EpiEstim vignette, which intends to provide a greater range of examples of using EpiEstim in practice.[84] A suggestion to improve usability by providing a library of pre-built configurations for different pathogens has already begun to be addressed to some extent by the R package EpiNow2.[69]

This is a fast-moving field and novel extensions or applications of EpiEstim-like methods are continuously emerging. It is therefore likely that we have missed valuable modifications not covered by articles available at the time of our literature search. This includes an extension to the method by Johnson et al. to account for superspreading, and work by the authors of this study, for example, the development of multi-variant EpiEstim (MV-EpiEstim) to estimate the transmission advantage of new pathogen variants or strains in real-time.[85, 86] It is also possible that through using EpiEstim as a starting point for our literature search, we may have missed relevant papers that did not cite the method. However, given that EpiEstim has been recognised as the most reliable currently existing approach for real-time R_t_ estimation, [12] we believe it is unlikely that we would have missed important contributions that would alter our conclusions. To our knowledge, there are still numerous issues regarding real-time estimation of the reproduction number that remain unaddressed by R packages or opensource software. For instance, we found no readily available tools that consider non-spatial population heterogeneities or the ability to include time-varying generation times.[87, 88]

The intention of this review was to provide a broad overview of the current landscape of renewal-equation based R_t_ estimation methods and tools. Therefore, beyond providing an appraisal of the usability of each identified R package/tool in terms of ease of installation, available documentation, and speed, we have not tested or compared their performance. Future work should focus on a more systematic and critical evaluation of the estimation accuracy of R packages intending to tackle the same issues. Moreover, it would be interesting to characterise the added value of additional features in relation to their cost, such as longer computational time.

## Conclusion

The quality of incidence data continues to pose significant challenges to real-time estimation of R_t_. Numerous methods and R packages have been developed to address some of these issues, but significant gaps remain, such as the inability to directly use temporally aggregated incidence data. Despite the importance of spatial factors in transmission, no R package has been identified to account for movement or interactions between locations when estimating R_t_. It is also clear that extensions to these methods could allow for rapid translation of R_t_ estimates into logistically relevant outputs, such as predicting how the demand for hospital resources may change in real-time. Addressing these recurring issues and extending the methodology to directly answer important practical and logistical questions are key priorities for widening the applicability of R_t_ estimation methods during epidemics. However, package developers should keep in mind that speed, ease of use, and access to sufficient resources, will be key to the uptake of these new or improved tools.

## Supporting information

S1 Text Figure A. The disease or pathogen under investigation in A) the papers that used an unmodified version of the EpiEstim package or the method and B) the papers that used a modified version of the approach or package. The category “multiple” refers to papers where more than one disease or pathogen were investigated. Note the diseases are different in both panels. Table A. Summary table of the papers identified that used an unmodified version of EpiEstim (*n* = 242). Table B. Usability of each R package or tool* identified in the scoping review. This table shows a full breakdown of how the classifications (very good = ✓✓, good = ✓, poor = ✗) were determined for the “additional exploration” section of Table 2 within the main text. For the ‘ease of installation’ and ‘documentation and tutorials’ sections, each criterion was allocated a score, shown in squared brackets, and the overall classification was determined by the sum of the scores. For the ‘speed’ section, each author used the system.time() function in R to determine the run time of the main function of the package available in the provided examples. ** The classification (<10s = ✓✓, >10s – 5min = ✓, >5min = ✗) was decided based on the time category agreed on by at least 2 out of the 3 computers. Figure B. Map showing the country in which each questionnaire respondent is based. The majority of responses were from the USA (*n* = 4), followed by Canada (*n* = 2), France (*n* = 2) and Indonesia (*n* = 2). There was one response from each of Austria, Bermuda (circled), Germany, India, Peru, Uruguay, and the UK. Figure C. A) The profession of each questionnaire respondent and B) the purpose of their analysis. Respondents could select more than one answer for both questions. Figure D. A) Disease(s) investigated by each respondent. B) Categories of input data. Respondents could select more than one answer for both questions. Figure E. Broad reason for the use of EpiEstim. Respondents could select more than one answer for this question. Figure F. Questionnaire responses to A) how well the package met the needs of each respondent on a scale from 1 to 5 (1: “badly”, 5: “very well”), and B) “Which features do you think could be improved?”. Table C. Summary of the issues and suggestions reported in questionnaire feedback categorised by broad theme. *n* is the number of respondents and % is the percentage of the 17 respondents who reported or made a suggestion regarding the issue.
(PDF)
