# Supplementary material for: Real-time estimation of the epidemic reproduction number: Scoping review of the applications and challenges
Source: PLOS Digit Health. 2022 Jun 27;1(6):e0000052. doi: 10.1371/journal.pdig.0000052 (PMC9931334; doi:10.1371/journal.pdig.0000052)
Supplement: S1 Text — Figure A. The disease or pathogen under investigation in A) the papers that used an unmodified version of the EpiEstim package or the method and B) the papers that used a modified version of the approach or package. The category “multiple” refers to papers where more than one disease or pathogen were investigated. Note the diseases are different in both panels. Table A. Summary table of the papers identified that used an unmodified version of EpiEstim (n = 242). Table B. Usability of each R package or tool* identified in the scoping review. This table shows a full breakdown of how the classifications (very good = ✓✓, good = ✓, poor = ✗) were determined for the “additional exploration” section of Table 2 within the main text. For the ‘ease of installation’ and ‘documentation and tutorials’ sections, each criterion was allocated a score, shown in squared brackets, and the overall classification was determined by the sum of the scores. For the ‘speed’ section, each author used the system.time() function in R to determine the run time of the main function of the package available in the provided examples. ** The classification (<10s = ✓✓, >10s – 5min = ✓, >5min = ✗) was decided based on the time category agreed on by at least 2 out of the 3 computers. Figure B. Map showing the country in which each questionnaire respondent is based. The majority of responses were from the USA (n = 4), followed by Canada (n = 2), France (n = 2) and Indonesia (n = 2). There was one response from each of Austria, Bermuda (circled), Germany, India, Peru, Uruguay, and the UK. Figure C. A) The profession of each questionnaire respondent and B) the purpose of their analysis. Respondents could select more than one answer for both questions. Figure D. A) Disease(s) investigated by each respondent. B) Categories of input data. Respondents could select more than one answer for both questions. Figure E. Broad reason for the use of EpiEstim. Respondents could select more than one answer for this que [file pdig.0000052.s001.pdf]

Supplementary Information:
Real-time estimation of the epidemic reproduction number: scoping review of the applications and challenges

Rebecca K Nash[1] MSc, Pierre Nouvellet[1,2] PhD*, Anne Cori[1] PhD*

*Contributed equally

[1]*MRC Centre for Global Infectious Disease Analysis, Jameel Institute, School of Public Health, Imperial College London*

[2]*School of Life Sciences, University of Sussex*

Contents

1. Overview....1
2. Scoping Review: additional results....1
3. Exploration of R package and tool usability....2
4. Questionnaire: additional results....7
5. Questionnaire....11
6. References....16

1. Overview

In this supplementary information, we provide more detailed results from our scoping review, including additional exploration to determine the usability of each identified R package and tool, more in depth analyses of our questionnaire data, and the questionnaire itself.

2. Scoping Review: additional results

Our scoping review identified 281 papers that used an unmodified (*n*=242) or modified (*n*=54) version of EpiEstim. There were a variety of diseases under investigation, but the most common were COVID-19, followed by Ebola Virus Disease and Influenza (Figure A).

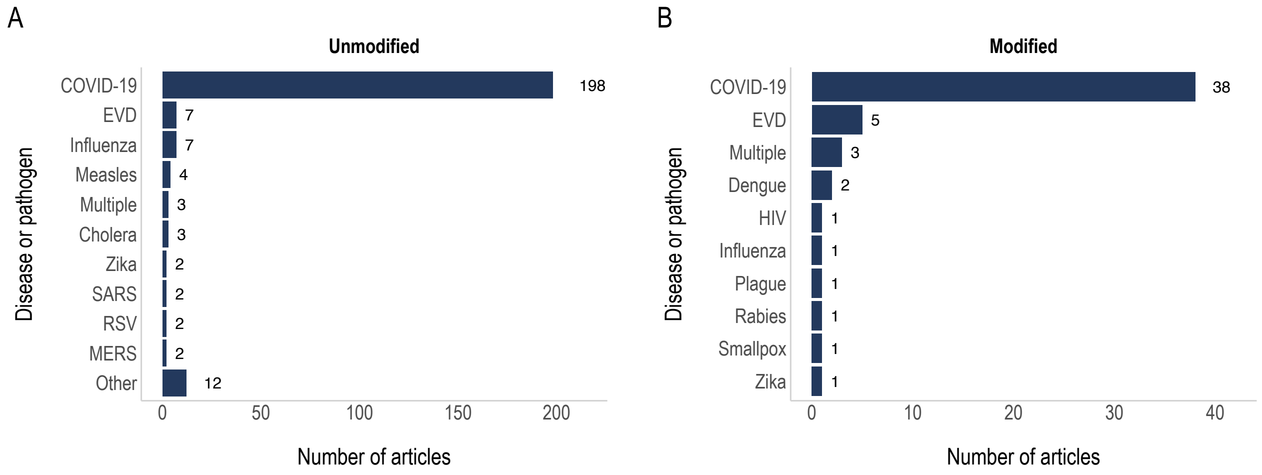

Figure A. The disease or pathogen under investigation in A) the papers that used an unmodified version of the EpiEstim package or the method and B) the papers that used a modified version of the approach or package. The category "multiple" refers to papers where more than one disease or pathogen were investigated. Note the diseases are different in both panels.

Between 2013 and December 2020, 242 papers were identified that used an unmodified version of EpiEstim or the underlying method (Table A). The majority (82%) of these papers were investigating COVID-19 (*n*=198).

Table A. Summary table of the papers identified that used an unmodified version of EpiEstim (*n*=242).

Disease/disease agent investigated	*n*	References
COVID-19	198	1-3, 5-8, 13-23, 25-32, 37-39, 42-46, 48-66, 70-74, 76-85, 89-102, 106, 107, 109, 111-113, 115-125, 127-140, 143, 145-147, 149-152, 154-157, 159-179, 181-197, 199-202, 204-213, 215, 216, 220, 223-226, 228-241
Ebola Virus Disease (EVD)	7	9, 68, 103, 108, 110, 203, 227
Influenza	7	10, 11, 40, 69, 75, 114, 180
Measles	4	86, 87, 88, 221
Multiple diseases	3	35, 141, 217
Cholera	3	24, 33, 218
Zika	2	142, 153
Severe Acute Respiratory Syndrome (SARS)	2	36, 104
Respiratory Syncytial Virus (RSV)	2	12, 144
Middle East Respiratory Syndrome (MERS)	2	148, 214
Other	12	4, 34, 41, 47, 67, 105, 126, 158, 198, 219, 222, 242

3. Exploration of R package and tool usability

We performed some additional exploration to determine the usability of each R package and tool (Table B & Table 2 in the main text). This was assessed by the ease of installation, the availability of adequate documentation or tutorials, and their speed. Table B provides the full breakdown for how each score was defined.

<u>Ease of installation</u>

Ease of installation was determined by the availability of installation instructions, the options for how the R package could be installed, the R package dependencies, and whether other system dependencies were required for either running or installing the packages. As EpiFilter is not an R package, some of the criteria in this section are not applicable. To use EpiFilter, the repository needs to be cloned from GitHub.[243]

Installation Instructions

Installation instructions may be available through an adequate README file or website, e.g. a "quick start" or introductory page. Instructions were found for all R packages except for APEestim and bayEStim.

Options for installation

Installation is easiest if the R package is available on the Comprehensive R Archive Network (CRAN), by simply using the "install.packages()" function. earlyR, epicontacts and EpiNow2 are available on CRAN. All remaining R packages can be installed using the devtools R package, which enable packages to be installed and built directly from source (e.g.

GitHub).[244,245] If the package has compiled code, as is the case for Epidemia, then devtools requires that the user has a working development environment (e.g. Rtools for Windows and Xcode for Mac).

R package dependencies

R package dependencies have the potential to complicate installation, particularly if there are many dependencies or if package versions are specified. For example, certain packages and/or package versions may not be compatible with the version of R that the user has installed. The chance of issues arising increases with the greater number of package dependencies, but we chose 10 dependencies as an arbitrary cut off for the usability score.

Additional system dependencies required for running or installing the packages

Three of the packages: bayEStim, Epidemia, and EpiNow2, require additional system (or software) dependencies, in addition to R packages. bayEStim depends on the R package rjags to run, which itself requires users to download and install the programme JAGS (Just Another Gibbs Sampler).[246,247] As bayEStim has no documentation, this process is not clear, especially for less experienced users. Epidemia and EpiNow2 use the Stan probabilistic programming language and depend on the R package rstan, which provides an R interface that allows Stan models to be fit.[248,249] To install rstan, users are required to install a recent C++ compiler and configure R to use it. Both Epidemia and EpiNow2 provide installation instructions including links to the rstan wiki.[250]

Documentation and tutorials

We have determined whether each R package or tool has function documentation, a vignette or worked examples. At a minimum, function documentation including details on function arguments and outputs should be made available. APEstim and bayEStim only provide function documentation, but no examples, which would make both difficult to use. All the remaining packages and tool have a vignette (or an equivalent, such as a webpage with tutorials) with one or more worked examples.

Speed

Computational speed has been assessed by each author of this study with different computer specifications (see Table B). The main function in each package/tool was taken from provided examples (if available) and wrapped in the system.time() R function to measure the execution time. The main function estimated the reproduction number for each R package/tool except for epicontacts, which estimates the serial interval. We chose the following classifications: <10 seconds = very good, 10 seconds – 5 minutes = good, >5 minutes = poor. The classification allocated to each package was based on the agreement of at least 2 out of the 3 computers. We note that such direct comparisons of the runtimes of the different models may not be fair, as the examples provided by each package which we have used to assess speed vary in terms of the dataset used, model complexity, and dimensionality of the reproduction number to estimate. Nevertheless, we assume that examples will always be relatively simple and therefore their computational speed may be a good overall indicator of speed of reproduction number estimation in general using a given package.

In our experience, of those that estimated the reproduction number, the fastest packages were APEestim, earlyR and EpiFilter, all taking <10 seconds. Epidemia took from <1 minute to nearly 4 minutes, depending on whether the model was basic (uses renewal equation to propagate infections) or extended (adds variance to this process). Interestingly, the extended model took less time to run, which as discussed on the package website, may be due to the posterior distribution being easier to sample from.[251] Meanwhile, estimating the reproduction number using the epinow() function in EpiNow2 took longer, from 3 up to 13 minutes. This took much longer when estimating the reproduction number for each geographical

region in turn using regional_epinow(), ranging from 8 to 25 minutes. Both Epidemia and EpiNow2 give an indication of progress whilst estimation is ongoing. This is particularly important for EpiNow2 given the lengthy execution time, but the progress bar only refreshes after long intervals, so the user is not updated regularly.

Table B. Usability of each R package or tool* identified in the scoping review. This table shows a full breakdown of how the classifications (very good=✓✓, good=✓, poor=✗) were determined for the "additional exploration" section of Table 2 within the main text. For the 'ease of installation' and 'documentation and tutorials' sections, each criterion was allocated a score, shown in squared brackets, and the overall classification was determined by the sum of the scores. For the 'speed' section, each author used the system.time() function in R to determine the run time of the main function of the package available in the provided examples.** The classification (<10s = ✓✓, >10s – 5min = ✓, >5min = ✗) was decided based on the time category agreed on by at least 2 out of the 3 computers.

Theme	Usability criterion	**APEestim** *(v 0.0.1)*	**bayEStim** *(v 0.0.1)*	**earlyR** *(v 0.0.5)*	**epicontacts** *(v 1.1.2)*	**Epidemia** *(v 1.0.0)*	**EpiFilter*** *(*Tool)*	**EpiNow2** *(v 1.3.2)*
Ease of installation	Installation instructions available (e.g., detailed README file or webpage) **[1]**	0	0	1	1	1	0	1
	Installation via CRAN **[1]**	0	0	1	1	0	NA	1
	Installation via devtools **[1]**<br><br>*(Note: devtools may require the installation of Rtools (Windows) or Xcode (Mac))*	1	1	1	1	1	NA	1
	Number of package dependencies **[if n<10 score = 1]**	1 (n=0)	1 (n=3)	1 (n=5)	1 (n=7)	0 (n=18)	1 (n=0)	0 (n=23)
	Separate pre-requisite installations or configurations required outside of R **[if no pre-requisites, score = 1]**	1	0 (JAGS 4.x.y (rjags))	1	1	0 (Configure C++ toolchain (rstan))	NA (Download github repository)	0 (Configure C++ toolchain (rstan))
	**Overall ease of installation** Scoring:<br>[4-5] = ✓✓<br>[2-3] = ✓<br>[0-2] = ✗	✓	✗	✓✓	✓✓	✗	✗+	✓
Documentation and tutorials	Function documentation (description of inputs and outputs) **[1]**	1	1	1	1	1	1	1
	Vignette available (either included within the package or online) **[1]**	0	0	1	1	1	1	1

	Worked example(s) available **[1]**	0	0	1	1	1	1	1
	**Overall documentation and tutorials**<br>Scoring:<br>[3] = ✓✓<br>[2] = ✓<br>[0-1] = ✗	✗	✗	✓✓	✓✓	✓✓	✓✓	✓✓
**Speed**	Speed of estimation (estimation of the reproduction number for all except for epicontacts, which estimates the serial interval)<br><br>Based on provided examples** and the system.time() function on three computers (C1-3) with the following specifications:<br>**C1:** MacOS (2 GHz Quad-Core Intel Core i5) 16GB RAM<br>**C2:** MacOS (M1) 32GB RAM<br>**C3:** Windows (i7) 16GB RAM	apeEstim()<br><br>(Taken from EpiFilter vignette)<br><br>*Flu example*<br>C1: 0.227s<br>C2: 0.118s<br>C3: 0.19s<br><br>*SARS example*<br>C1: 0.206s<br>C2: 0.106s<br>C3: 0.22s	NA<br><br>(No examples provided)	get_R()<br><br>C1: 0.022s<br>C2: 3s<br>C3: 0.02s	get_pairwise()<br><br>C1: 0s<br>C2: 0s<br>C3: 0s	epim()<br><br>Basic model (fm1)<br><br>C1: 2m 29s<br>C2: 51s<br>C3: 2m 17s<br><br>Extended model (fm2)<br><br>C1: 43s<br>C2: 17s<br>C3: 52s	epiFilter()<br><br>*Flu example*<br>C1: 0.053s<br>C2: 0.022s<br>C3: 0.24s<br><br>*SARS example*<br>C1: 0.018s<br>C2: 0.007s<br>C3: 0.22s	epinow()<br><br>C1: 7m 50s<br>C2: 3m 22s<br>C3: 13m 23s<br><br>regional_epinow()<br><br>C1: 16m 36s<br>C2: 7m 47s<br>C3: 25m 02s
	**Overall speed**<br>Scoring based on what the majority of C1-3 found:<br><10s = ✓✓<br>>10s – 5min = ✓<br>>5min = ✗	✓✓	NA	✓✓	✓✓	✓++	✓✓	✗++

+ = Given that EpiFilter is not an R package, some of the criteria were not applicable. Given that it requires cloning a github repository, we have scored it with an ✗.

++ = function includes a progress bar or some other indication of progress

**Source of examples:

APEestim and EpiFilter: https://github.com/kpzoo/EpiFilter/blob/master/R%20files/vignette.R
earlyR: https://cran.r-project.org/web/packages/earlyR/vignettes/earlyR.html
epicontacts: https://cran.r-project.org/web/packages/epicontacts/vignettes/epicontacts.html
Epidemia: https://imperialcollegelondon.github.io/epidemia/articles/flu.html
EpiNow2: https://github.com/epiforecasts/EpiNow2

4. Questionnaire: additional results

The full questionnaire is shown in the next section. In total, there were 17 responses to the questionnaire from respondents in 11 different countries, including the USA, Canada, France, Indonesia, Austria, Bermuda, Germany, India, Peru, Uruguay, and the UK (Figure B). In an attempt to reach a wide variety of people, the questionnaire was distributed to a contact list of known EpiEstim users, in addition to being shared via the Imperial MRC Centre for Global Infectious Disease Analysis Twitter account (with over 141,000 followers). Nonetheless, the number of respondents was small, and it is possible that the sample may be more representative of epidemiologists, or those with a high level of training in the field, as opposed to other researchers who may experience greater barriers in terms of usability. Despite these limitations, the questionnaire helped us to limit the risk of publication bias and was useful to reinforce findings from our literature review whilst ensuring that we didn't overlook any issues that would be less apparent from the literature search alone.

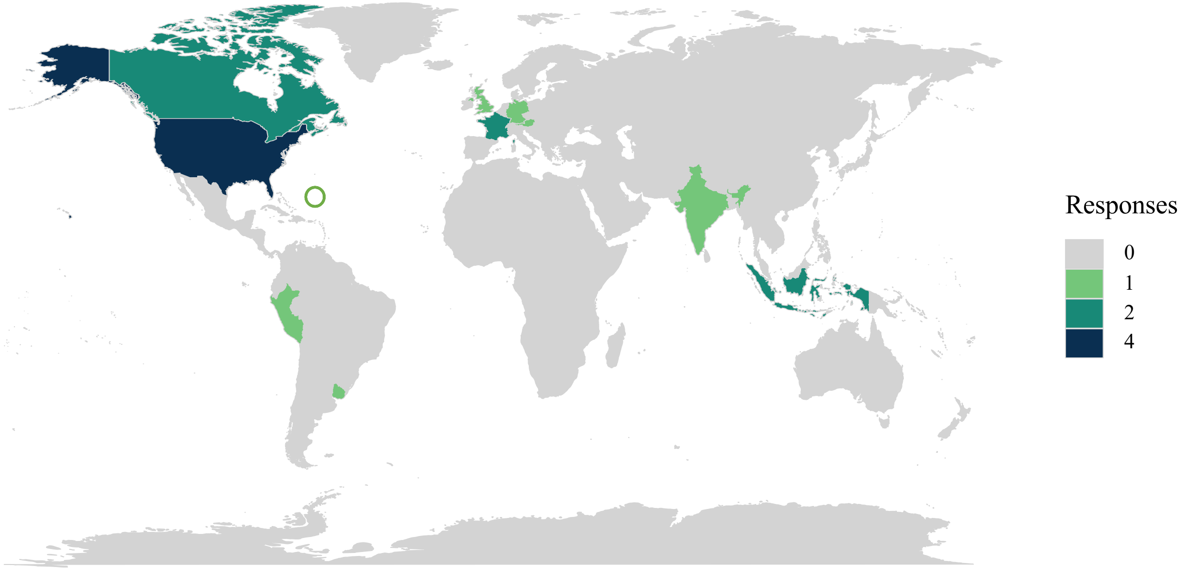

Figure B. Map showing the country in which each questionnaire respondent is based. The majority of responses were from the USA (*n*=4), followed by Canada (*n*=2), France (*n*=2) and Indonesia (*n*=2). There was one response from each of Austria, Bermuda (circled), Germany, India, Peru, Uruguay, and the UK.

Most respondents were academic researchers, public health consultants or health professionals (Figure C). The vast majority (94.1%) used EpiEstim for the purpose of real-time estimation of the reproduction number. Over half (58.8%) had used EpiEstim to retrospectively estimate the reproduction number, whilst the purpose of the analysis was less frequently for real-time incidence forecasting (18%) (Figure C). One respondent used EpiEstim for model calibration.

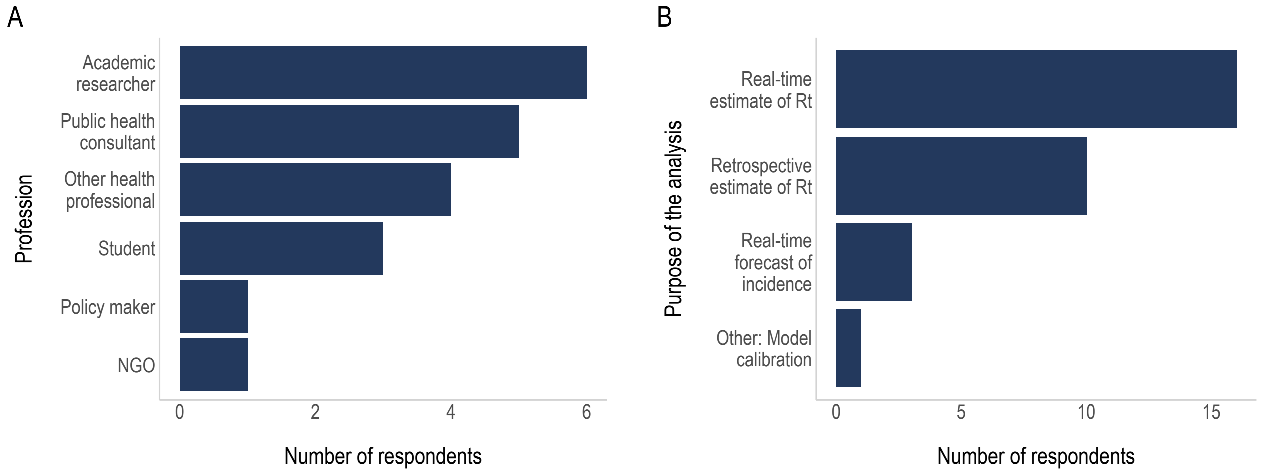

Figure C. A) The profession of each questionnaire respondent and B) the purpose of their analysis. Respondents could select more than one answer for both questions.

All questionnaire respondents had performed analysis using EpiEstim to investigate COVID-19 and some also used it for Ebola Virus Disease, Norovirus and Influenza (Figure D). In terms of input datasets, all respondents had access to daily case incidence data, 24% had daily death data, and 12% had weekly case incidence data (Figure D). Only one respondent reported having data that distinguished between local and imported cases and none reported having data containing infector-infected pairs.

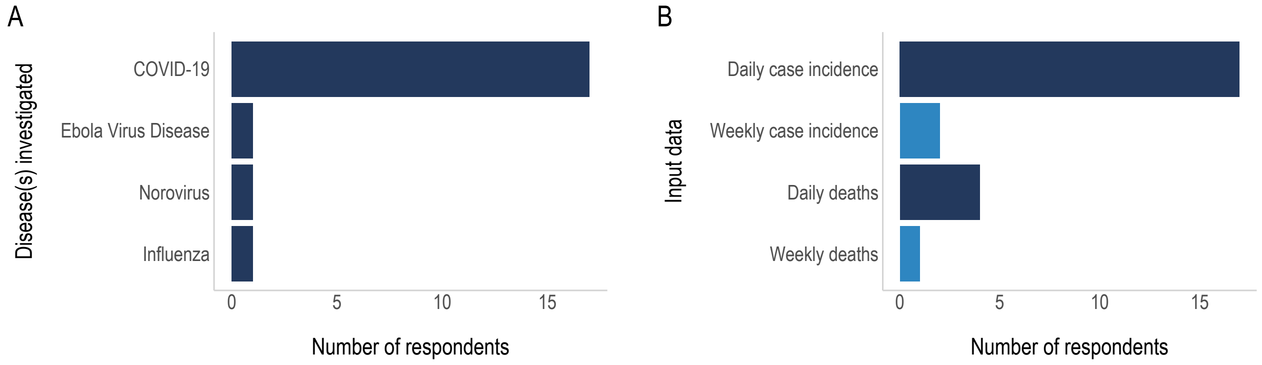

Figure D. A) Disease(s) investigated by each respondent. B) Categories of input data. Respondents could select more than one answer for both questions.

The analyses predominantly informed academic research (53%), intervention planning (35%), governmental situation reports (29%) and logistical planning (29%) (Figure E).

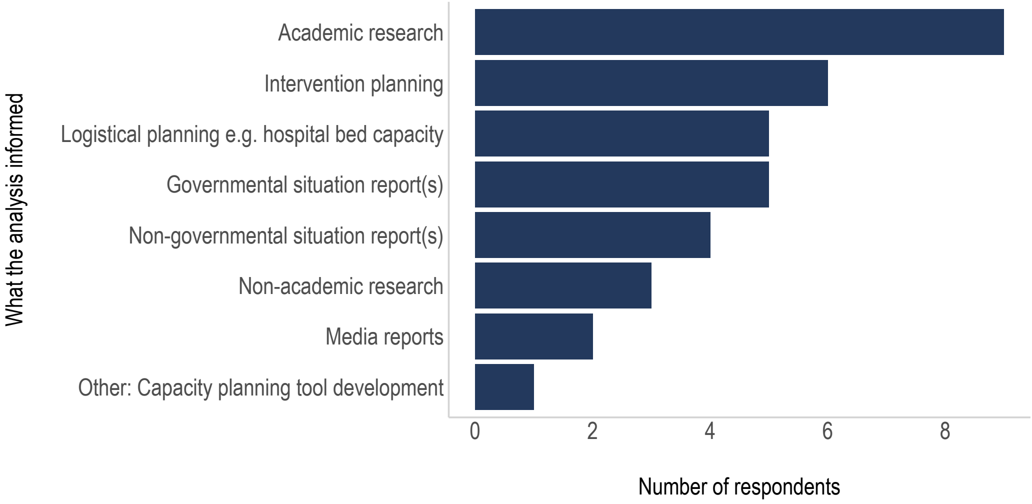

Figure E. Broad reason for the use of EpiEstim. Respondents could select more than one answer for this question.

On a scale from 1 to 5 (1 representing "badly" and 5 "very well") respondents were asked how well EpiEstim met their needs. All respondents selected between 3 and 5, with 94% selecting either 4 or 5 (Figure F). Despite this, twelve of the respondents (71%) reported having a technical or methodological issue when using EpiEstim.

Almost half of respondents (47%) said they thought none of EpiEstim's features could be improved, whereas 35% thought that usability could be improved (Figure F). One respondent (6%) thought speed could be improved and 24% selected "other" and mentioned either issues understanding how to use the package properly (n=2), wanting extra features (n=1) or issues with compatibility (n=1).

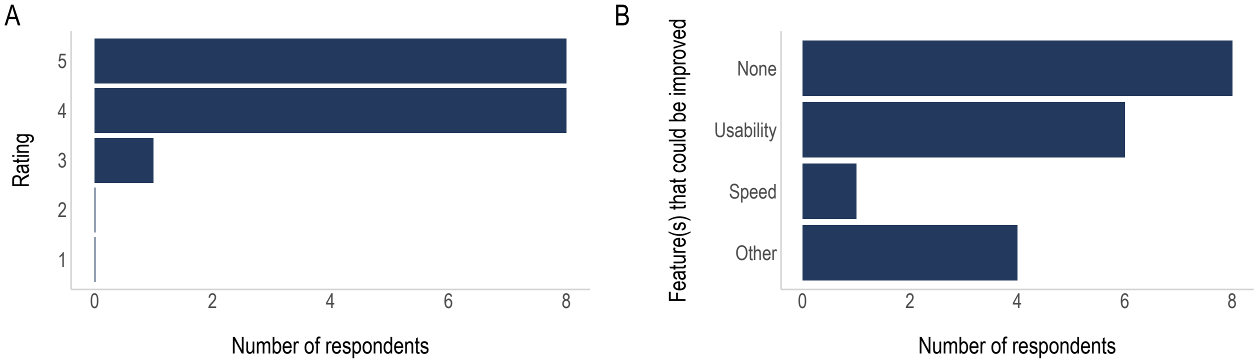

Figure F. Questionnaire responses to A) how well the package met the needs of each respondent on a scale from 1 to 5 (1: "badly", 5: "very well"), and B) "Which features do you think could be improved?".

Although when asked directly, only 35% of respondents said usability was an issue (Figure F), users' responses were classified as such (Table C) if they reported an issue that suggested a lack of understanding of the package. For example, one respondent reported a technical issue as being unable to estimate R_t for day 1 of the outbreak, but EpiEstim can only start estimating R_t on at least day 2 because it requires data on past incidence.[252]

The questionnaire highlighted similar challenges to the scoping review, including issues with incidence data, geographical factors and suggestions to extend the capability of the package to address practical and logistical issues (Table C). Respondents wanted to account for reporting delays and time-varying reporting rates, to use weekly as well as daily incidence, different formats of data, and more intuitive plotting options for the results. Regarding geographical issues, they suggested enabling easier reproduction number estimates for different regions simultaneously. For more practical purposes, they proposed expanding the methodology to allow for the projection of hospital bed occupancy, thereby assisting logistical planning in hospitals. As discussed in the main text, the questionnaire also revealed an additional three themes: usability, speed, and compatibility.

It is important to note that, as shown in section 5, for four of the survey questions that required typed answers as opposed to multiple choice, we used example responses to ensure that questions were clear for respondents. These examples were based on issues previously reported in correspondence with users of EpiEstim. It is possible that these examples may have biased the results by making respondents more likely to state these particular issues compared to others.

Table C. Summary of the issues and suggestions reported in questionnaire feedback categorised by broad theme. *n* is the number of respondents and % is the percentage of the 17 respondents who reported or made a suggestion regarding the issue.

Theme	Issue	Suggestion	*n*	%
**Incidence data**	• Unable to account for reporting delays or reporting rates<br>• Cannot use weekly aggregated incidence data		2	12
**Geographical or spatial**		• Allow users to model different regions at the same time	1	6
**Practical or logistical applications**		• Extend capability to aid logistical planning in hospitals	1	6
**Usability**	• Issues understanding how to use the package properly	• Add a pre-processing function to help novice users when setting up parameters<br>• More resources with examples of EpiEstim implementation<br>• Provide more intuitive plotting options	9	53
**Speed**	• Slow when accounting for uncertainty in the serial interval distribution		3	18
**Compatibility**	• Unable to use certain software or alternative programming languages	• Examples of how EpiEstim can be used in a workflow with other R packages	3	18

5. Questionnaire

MRC Centre for Global Infectious Disease Analysis

Imperial College London

EpiEstim Feedback

The information collected in this survey is for academic purposes and will be used to inform EpiEstim package development. We are very grateful for any feedback you can provide.

The estimated time to complete the survey is 5-10 minutes.

* Required

Which version(s) of EpiEstim do you use? *

You can select more than one option.

- [ ] R package

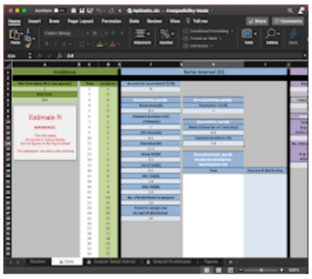

- [ ] Excel

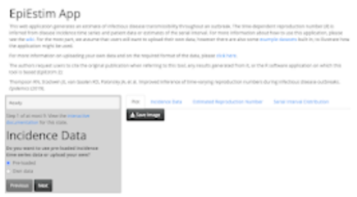

- [ ] Web Interface (Shiny app)
- [ ] Custom

Which category best describes you?

- [ ] Academic Researcher
- [ ] Policy Maker
- [ ] Public Health Consultant
- [ ] Clinician
- [ ] Other Health Professional
- [ ] Student
- [ ] Other: ______

Which country are you based in?

Your answer ______

What is/was the purpose of your analysis? *

You can select more than one option.

- [ ] Real-time estimate of the reproduction number (R)
- [ ] Retrospective estimate of the reproduction number (R)
- [ ] Real-time forecast of future case incidence
- [ ] Other: ______

What did this analysis inform?

You can select more than one option.

- [ ] Academic research
- [ ] Non-academic research
- [ ] Intervention planning
- [ ] Governmental situation report(s)
- [ ] Non-governmental situation report(s)
- [ ] Media reports
- [ ] Logistical planning e.g. hospital bed capacity
- [ ] Other: ______

Which disease(s) were/are you investigating? *

You can select more than one option.

- [ ] COVID-19
- [ ] Ebola Virus Disease
- [ ] Other: ______

Which of these categories apply to your input data set(s)? *

You can select more than one option.

- [ ] Daily incidence
- [ ] Weekly incidence
- [ ] Daily deaths
- [ ] Weekly deaths
- [ ] Data that distinguished between local and imported cases
- [ ] Data containing infector-infected pairs
- [ ] Other: ______

Overall, how well does the package meet your needs? *

	1	2	3	4	5	
Badly	◯	◯	◯	◯	◯	Very well

Have you encountered any technical issues using EpiEstim? If so, what were they? *

E.g. Excel spreadsheet imposes limit on daily incidence.

Your answer

Have you encountered any other methodological issues? If so, what were they? *

E.g. I want to use weekly incidence, not daily incidence.

Your answer

Which features do you think could be improved? *

You can select more than one option.

- [ ] Usability
- [ ] Speed
- [ ] None
- [ ] Other: ____________

Can you provide examples of issues you've had with respect to the features selected in the previous question?

E.g. I had issues with speed when I accounted for uncertainty in the serial interval. ("method=uncertain_si")

Your answer

If you had issues with speed, what were the parameters you used?

E.g. If using the R package, which arguments did you assign to the estimate_r() function? (Feel free to just copy and paste your code)

Your answer

Are there any additions/modifications that would improve the usability of the package? *

Your answer

Have you made any modifications yourself? If yes, what were they?

Your answer

Would you be willing to be contacted to discuss further or share your code with us? If so, please submit your email address here.*

*You are consenting to someone from the team following up in due course at this email address

Your answer

Submit

Never submit passwords through Google Forms.

This content is neither created nor endorsed by Google. Report Abuse - Terms of Service - Privacy Policy

Google Forms

6. References

1. Abbott S. Temporal variation in transmission during the COVID-19 outbreak in Italy [Internet]. CMMID Repository. 2020 [cited 2021 Jun 2]. Available from: https://cmmid.github.io/topics/covid19/italy-time-varying-transmission.html

2. Acuña-Zegarra MA, Santana-Cibrian M, Velasco-Hernandez JX. Modeling behavioral change and COVID-19 containment in Mexico: A trade-off between lockdown and compliance. Mathematical Biosciences. 2020 Jul 1;325:108370.

3. Adak D, Majumder A, Bairagi N. Mathematical perspective of Covid-19 pandemic: Disease extinction criteria in deterministic and stochastic models. Chaos Solitons Fractals. 2021 Jan;142:110381.

4. Adams C, Young D, Gastañaduy PA, Paul P, Marsh Z, Hall AJ, et al. Quantifying the roles of vomiting, diarrhea, and residents vs. staff in norovirus transmission in U.S. nursing home outbreaks. PLOS Computational Biology. 2020 Mar 25;16(3):e1007271.

5. Adegboye OA, Adekunle AI, Gayawan E. Early Transmission Dynamics of Novel Coronavirus (COVID-19) in Nigeria. International Journal of Environmental Research and Public Health. 2020 Jan;17(9):3054.

6. Adeyeri O, Oyekan K, Ige S, Akinbobola A, Okogbue E. Modelling COVID-19 cases in Nigeria: Forecasts, uncertainties, projections and the link with weather. Pre-print [Internet]. 2020 Jul 14 [cited 2021 Jun 2]; Available from: https://www.researchsquare.com

7. Agapiou S, Anastasiou A, Baxevani A, Christofides T, Constantinou E, Hadjigeorgiou G, et al. Modeling of Covid-19 Pandemic in Cyprus. arXiv:201001927 [stat] [Internet]. 2020 Oct 5 [cited 2021 Jun 2]; Available from: http://arxiv.org/abs/2010.01927

8. Aghaali M, Kolifarhood G, Nikbakht R, Saadati HM, Hashemi Nazari SS. Estimation of the serial interval and basic reproduction number of COVID-19 in Qom, Iran, and three other countries: A data-driven analysis in the early phase of the outbreak. Transbound Emerg Dis [Internet]. 2020 Jun 16 [cited 2021 Jun 2]; Available from: https://www.ncbi.nlm.nih.gov/pmc/articles/PMC7300937/

9. Ahmed T. Improving Disease Surveillance Programs in Liberia [Internet] [Thesis]. Johns Hopkins University; 2019 [cited 2021 Jun 2]. Available from: https://dspace-prod.mse.jhu.edu/handle/1774.2/62045

10. Ali ST, Kadi AS, Ferguson NM. Transmission dynamics of the 2009 influenza A (H1N1) pandemic in India: The impact of holiday-related school closure. Epidemics. 2013 Dec;5(4):157–63.

11. Ali ST, Wu P, Cauchemez S, He D, Fang VJ, Cowling BJ, et al. Ambient ozone and influenza transmissibility in Hong Kong. European Respiratory Journal [Internet]. 2018 May 1 [cited 2021 Jun 2];51(5). Available from: https://erj.ersjournals.com/content/51/5/1800369

12. Ali ST, Tam CC, Cowling BJ, Yeo KT, Yung CF. Meteorological drivers of respiratory syncytial virus infections in Singapore. Sci Rep. 2020 Nov 24;10(1):20469.

13. Ali ST, Wang L, Lau EHY, Xu X-K, Du Z, Wu Y, et al. Serial interval of SARS-CoV-2 was shortened over time by nonpharmaceutical interventions. Science. 2020 Aug 28;369(6507):1106–9.

14. Amrutha GS, Sharma A, Sharma A. A Comparative Study to Find a Suitable Model for an Improved Real-Time Monitoring of The Interventions to Contain COVID-19 Outbreak in The High Incidence States of India. medRxiv. 2020 Sep 15;2020.09.14.20190447.

15. Arnon A, Ricco J, Smetters K. Epidemiological and Economic Effects of Lockdown. Available from: https://www.brookings.edu/wp-content/uploads/2020/09/Arnon-et-al-conference-draft.pdf pp.53.

16. Arroyo Marioli F, Bullano F, Kučinskas S, Rondón-Moreno C. Tracking R of COVID-19: A New Real-Time Estimation Using the Kalman Filter. Available at SSRN 3581633. 2020;

17. Baibás LAB, Conesa MG, Caravaca GR, Baibás BB. COVID-19 effective reproductive ratio determination: An application, and analysis of issues and influential factors. medRxiv. 2020 Aug 12;2020.07.15.20154039.

18. Bandoy DDR, Weimer BC. Pandemic dynamics of COVID-19 using epidemic stage, instantaneous reproductive number and pathogen genome identity (GENI) score: modeling molecular epidemiology. medRxiv. 2020 Mar 20;2020.03.17.20037481.

19. Bao C, Pan E, Ai J, Dai Q, Xu K, Shi N, et al. COVID-19 outbreak following a single patient exposure at an entertainment site: an epidemiological study. Transboundary and Emerging Diseases. 2020;

20. Basu D, Salvatore M, Ray D, Kleinsasser M, Purkayastha S, Bhattacharyya R, et al. A comprehensive public health evaluation of lockdown as a non-pharmaceutical intervention on COVID-19 spread in India: National trends masking state level variations. medRxiv. 2020;

21. Baunez C, Degoulet M, Luchini S, Pintus ML, Pintus PA, Teschl M. The Acceleration Index as a Test-Controlled Reproduction Number: Application to COVID-19 in France. medRxiv. 2020 Dec 2;2020.12.01.20241570.

22. Bemanian A, Ahn KW, O'Brien M, Rausch DJ, Weston B, Beyer KM. Investigating the Trajectory of the COVID-19 Outbreak in Milwaukee County and Projected Effects of Relaxed Distancing. WMJ: official publication of the State Medical Society of Wisconsin. 2020;119(2):84–90.

23. Bhaduri R, Roy S, Pal SK. Rough-Fuzzy CPD: A Gradual Change Point Detection Algorithm. arXiv preprint arXiv:201006370. 2020;

24. Bi Q, Abdalla FM, Masauni S, Reyburn R, Msambazi M, Deglise C, et al. The epidemiology of cholera in Zanzibar: implications for the zanzibar comprehensive cholera elimination plan. The Journal of infectious diseases. 2018;218(suppl_3):S173–80.

25. Bibi MU, Aliyu SA, Jega IM. Analysing secondary infections of Coronavirus Disease 2019 across the Geopolitical zones of Nigeria using estimated time dependent reproduction number. Sriwijaya Journal of Environment. 2020;5(2):103–10.

26. Bittihn P, Hupe L, Isensee J, Golestanian R. Local measures enable COVID-19 containment with fewer restrictions due to cooperative effects. EClinicalMedicine [Internet]. 2021 Feb 1 [cited 2021 Jun 2];32. Available from: https://www.thelancet.com/journals/eclinm/article/PIIS2589-5370(20)30462-4/abstract

27. Blokh A, Penievskaya N, Rudakov N, Lazarev I. Epidemic potential of COVID-19 in Omsk region and assessment of the anti-epidemic measures. [Russian]. Fundamental and Clinical Medicine. 2020 Sep 29;5(3):8–17.

28. Bo Y, Guo C, Lin C, Zeng Y, Li HB, Zhang Y, et al. Effectiveness of non-pharmaceutical interventions on COVID-19 transmission in 190 countries from 23 January to 13 April 2020. Int J Infect Dis. 2021 Jan;102:247–53.

29. Bod'ová K, Boža V, Brejová B, Kollár R, Mikušová K, Vinař T. Time-adjusted Analysis Shows Weak Associations Between BCG Vaccination Policy and COVID-19 Disease Progression. medRxiv. 2020 May 6;2020.05.01.20087809.

30. Born B, Dietrich AM, Müller GJ. The lockdown effect: A counterfactual for Sweden. PLOS ONE. 2021 Apr 8;16(4):e0249732.

31. Bryant P, Elofsson A. The effect of opening up the US on COVID-19 spread. medRxiv. 2020 Jul 4;2020.07.03.20145649.

32. Caicedo-Ochoa Y, Rebellón-Sánchez DE, Peñaloza-Rallón M, Cortés-Motta HF, Méndez-Fandiño YR. Effective Reproductive Number estimation for initial stage of COVID-19 pandemic in Latin American Countries. International Journal of Infectious Diseases. 2020 Jun 1;95:316–8.

33. Camacho A, Bouhenia M, Alyusfi R, Alkohlani A, Naji MAM, Radiguès X de, et al. Cholera epidemic in Yemen, 2016–18: an analysis of surveillance data. The Lancet Global Health. 2018 Jun 1;6(6):e680–90.

34. Campbell EM, Jia H, Shankar A, Hanson D, Luo W, Masciotra S, et al. Detailed Transmission Network Analysis of a Large Opiate-Driven Outbreak of HIV Infection in the United States. J Infect Dis. 2017 Nov 1;216(9):1053–62.

35. Campbell F, Strang C, Ferguson N, Cori A, Jombart T. When are pathogen genome sequences informative of transmission events? PLOS Pathogens. 2018 Feb 8;14(2):e1006885.

36. Campbell F, Cori A, Ferguson N, Jombart T. Bayesian inference of transmission chains using timing of symptoms, pathogen genomes and contact data. PLOS Computational Biology. 2019 Mar 29;15(3):e1006930.

37. Canals M, Cuadrado C, Canals A, Yohannessen K, Lefio LA, Bertoglia MP, et al. Epidemic trends, public health response and health system capacity: the Chilean experience in four months of the COVID-19 pandemic. Rev Panam Salud Publica [Internet]. 2020 Aug 17 [cited 2021 Jun 2];44. Available from: https://www.ncbi.nlm.nih.gov/pmc/articles/PMC7429930/

38. Canals M. Informe Covid 19. Chile al 03/05/2020 - Escuela de Salud Pública - Universidad de Chile [Internet]. [cited 2021 Jun 2]. Available from: http://www.saludpublica.uchile.cl/noticias/163053/informe-covid-19-chile-al-03052020

39. Canals M. Report Covid 19. Chile to 04/25/2020 - School of Public Health - University of Chile [Internet]. [cited 2021 Jun 2]. Available from: http://www.saludpublica.uchile.cl/noticias/162949/informe-covid-19-chile-al-25042020

40. Carey J. Characterizing risk of transmission of respiratory viruses among school children using proxies of infectious contact and genetic data [Internet] [Thesis]. Johns Hopkins University; 2016 [cited 2021 Jun 2]. Available from: https://dspace-prod.mse.jhu.edu/handle/1774.2/39486

41. Carter-Gates M, Balestreri C, Thorpe SE, Cottier F, Baylay A, Bibby TS, et al. Implications of increasing Atlantic influence for Arctic microbial community structure. Sci Rep. 2020 Nov 6;10(1):19262.

42. Cassidy-Bushrow AE, Baseer M, Kippen K, Levin AM, Li J, Loveless I, et al. Social distancing during the COVID-19 pandemic: quantifying the practice in Michigan – a "hotspot state" early in the pandemic – using a volunteer-based online survey. BMC Public Health. 2021 Jan 29;21(1):245.

43. Challen R, Brooks-Pollock E, Tsaneva-Atanasova K, Danon L. Meta-analysis of the SARS-CoV-2 serial interval and the impact of parameter uncertainty on the COVID-19 reproduction number. medRxiv. 2020 Nov 20;2020.11.17.20231548.

44. Challen R, Tsaneva-Atanasova K, Pitt M, Edwards T, Gompels L, Lacasa L, et al. Estimates of regional infectivity of COVID-19 in the United Kingdom following imposition of social distancing measures. medRxiv. 2020 Nov 13;2020.04.13.20062760.

45. Chamberlain SD, Singh I, Ariza C, Daitch A, Philips P, Dalziel BD. Real-time detection of COVID-19 epicenters within the United States using a network of smart thermometers. medRxiv. 2020 Apr 10;2020.04.06.20039909.

46. Chan Y-WD, Flasche S, Lam T-LT, Leung M-HJ, Wong M-L, Lam H-Y, et al. Transmission dynamics, serial interval and epidemiology of COVID-19 diseases in Hong Kong under different control measures. Wellcome Open Res. 2020 Nov 9;5:91.

47. Chaves LF, Huber JH, Rojas Salas O, Ramírez Rojas M, Romero LM, Gutiérrez Alvarado JM, et al. Malaria Elimination in Costa Rica: Changes in Treatment and Mass Drug Administration. Microorganisms [Internet]. 2020 Jun 30 [cited 2021 Jun 2];8(7). Available from: https://www.ncbi.nlm.nih.gov/pmc/articles/PMC7409053/

48. Chaves LF, Hurtado LA, Rojas MR, Friberg MD, Rodríguez RM, Avila-Aguero ML. COVID-19 basic reproduction number and assessment of initial suppression policies in Costa Rica. Math Model Nat Phenom. 2020;15:32.

49. Chen T, Huang S, Li G, Zhang Y, Li Y, Zhu J, et al. Quantitative Effects of Entry Restrictions and Travel Quarantine on the Next Wave of COVID-19: Case Studies of China and Singapore. SSRN Journal [Internet]. 2020 [cited 2021 Jun 2]; Available from: https://www.ssrn.com/abstract=3622386

50. Cheng Q, Liu Z, Cheng G, Huang J. Heterogeneity and effectiveness analysis of COVID-19 prevention and control in major cities in China through time-varying reproduction number estimation. Sci Rep. 2020 Dec 15;10(1):21953.

51. Chintalapudi N, Battineni G, Sagaro GG, Amenta F. COVID-19 outbreak reproduction number estimations and forecasting in Marche, Italy. International Journal of Infectious Diseases. 2020 Jul 1;96:327–33.

52. Chong KC, Cheng W, Zhao S, Ling F, Mohammad KN, Wang MH, et al. Monitoring disease transmissibility of 2019 novel coronavirus disease in Zhejiang, China. Int J Infect Dis. 2020 Jul;96:128–30.

53. Chong KC, Cheng W, Zhao S, Ling F, Mohammad KN, Wang M, et al. Transmissibility of coronavirus disease 2019 in Chinese cities with different dynamics of imported cases. PeerJ [Internet]. 2020 Nov 6 [cited 2021 Jun 2];8. Available from: https://www.ncbi.nlm.nih.gov/pmc/articles/PMC7651459/

54. Cibrian MS, Velasco-Hernandez J, Corona-Moreno R. Reporte al 7 de Septiembre de 2020 de la estimación del semáforo epidemiológico de Querétaro. 2020 Sep.

55. Corona-Moreno R, Gonzalez N, Arguedas J, Velasco-Hernandez J. Nowcasting para el número reproductivo instantáneo Rt para la República Mexicana, la Ciudad de México, sus alcaldías, algunas ciudades y estados de México usando datos hasta el 08 de agosto [Internet]. Nodo Multidisciplinario de Matematicas Aplicadas: Instituto

de Matematicas UNAM Juriquilla; 2020 Sep. Available from: https://www.researchgate.net/publication/344178938_Nowcasting_R_t_CDMX_15Agosto

56. Corona-Moreno R, Gonzalez N, Velasco-Hernandez J. Tendencias de Rts de la epidemia de COVID-19 en México: reporte 20 de septiembre 2020 [Internet]. Nodo Multidisciplinario de Matematicas Aplicadas: Instituto de Matematicas UNAM Juriquilla; 2020 Sep. Available from: https://www.researchgate.net/profile/Jorge-Velasco-Hernandez/publication/344347054_Rts_CDMX_Edos_Ciudades_06Septiembre_Reported_21Septiembre/links/5f6ab61192851c14bc8e2eb9/Rts-CDMX-Edos-Ciudades-06Septiembre-Reported-21Septiembre.pdf

57. Corona-Moreno R, Gonzalez N, Velasco-Hernandez J. Tendencias de Rts de la epidemia de COVID-19 en México: reporte 05 de octubre 2020 [Internet]. Nodo Multidisciplinario de Matematicas Aplicadas: Instituto de Matematicas UNAM Juriquilla; 2020 Oct. Available from: https://www.researchgate.net/publication/344526176_Rts_CDMX_Edos_Ciudades_19Septiembre_Reported_05Octubre

58. Corona-Moreno R, Gonzalez N, Velasco-Hernandez J. Tendencias de la epidemia de COVID-19 en México: reporte 18 de octubre 2020 [Internet]. Nodo Multidisciplinario de Matematicas Aplicadas: Instituto de Matematicas UNAM Juriquilla; 2020 Oct. Available from: https://www.researchgate.net/profile/Jorge-Velasco-Hernandez/publication/344814471_Rts_CDMX_Edos_Ciudades_04Octubre_Reportado_19Octubre/links/5f919c0292851c14bcde7bb2/Rts-CDMX-Edos-Ciudades-04Octubre-Reportado-19Octubre.pdf

59. Corona-Moreno R, Gonzalez N, Velasco-Hernandez J. Tendencias de la epidemia de COVID-19 en México: reporte 9 de noviembre 2020 [Internet]. Nodo Multidisciplinario de Matematicas Aplicadas: Instituto de Matematicas UNAM Juriquilla; 2020 Nov. Available from: https://www.researchgate.net/publication/345815689_Rts_CDMX_Edos_Ciudades_24Octubre_Reported_09Noviembre

60. Corona-Moreno R, Gonzalez-Morales N, Arguedas J, Velasco-Hernandez J. Tendencias de Rts de la epidemia de COVID-19 en México: reporte 31 de agosto 2020 [Internet]. Nodo Multidisciplinario de Matematicas Aplicadas: Instituto de Matematicas UNAM Juriquilla; 2020 Sep. Available from: https://www.researchgate.net/publication/344233508_Rts_CDMX_Edos_Ciudades_29Agosto_Reported_13Septiembre

61. Corona-Moreno R, Santana Cibrian M, Velasco-Hernandez J. Reporte al 19 de septiembre de 2020 de la estimación del semáforo epidemiológico de Querétaro [Internet]. Nodo Multidisciplinario de Matematicas Aplicadas: Instituto de Matematicas UNAM Juriquilla; 2020 Sep. Available from: https://www.researchgate.net/publication/344347160_Reporte_al_19_de_septiembre_de_2020_de_la_estimacion_del_semaforo_epidemiologico_de_Queretaro

62. Corona-Moreno R, Santana Cibrian M, Velasco-Hernandez J. Reporte al 5 de octubre de 2020 de la estimación del semáforo epidemiológico de Querétaro [Internet]. Nodo Multidisciplinario de Matematicas Aplicadas: Instituto de Matematicas UNAM Juriquilla; 2020 Oct. Available from: https://www.researchgate.net/publication/344526419_Reporte_al_5_de_octubre_de_2020_de_la_estimacion_del_semaforo_epidemiologico_de_Queretaro

63. Corona-Moreno R, Santana Cibrian M, Velasco-Hernandez J. Estimación del semáforo epidemiológico de Querétaro: Reporte al 19 de octubre de 2020. Nodo Multidisciplinario de Matematicas Aplicadas: Instituto de Matematicas UNAM Juriquilla; 2020 Oct.

64. Cuadrado C, Monsalves MJ, Gajardo J, Bertoglia MP, Nájera M, Alfaro T, et al. Impact of small-area lockdowns for the control of the COVID-19 pandemic. medRxiv. 2020 May 9;2020.05.05.20092106.

65. Cui T, Yang G, Ji L, Zhu L, Zhen S, Shi N, et al. Chinese Residents' Perceptions of COVID-19 During the Pandemic: Online Cross-sectional Survey Study. J Med Internet Res [Internet]. 2020 Nov 25 [cited 2021 Jun 2];22(11). Available from: https://www.ncbi.nlm.nih.gov/pmc/articles/PMC7690970/

66. Čvokić D. Анализа ситуације и предвиђања у вези са епидемијом САРС-КоВ-2 вируса у Републици Српској на дан 27. мај 2020. [Serbian] [Internet]. OSF Preprints; 2020 [cited 2021 Jun 3]. Available from: https://osf.io/4yrtu/

67. Dai C, ZhiWang, Wang W, Li Y, Wang K, Dai C, et al. Epidemics and underlying factors of multiple-peak pattern on hand, foot and mouth disease inWenzhou, China. MBE. 2019;16(4):2168–88.

68. Dalziel BD, Lau MSY, Tiffany A, McClelland A, Zelner J, Bliss JR, et al. Unreported cases in the 2014-2016 Ebola epidemic: Spatiotemporal variation, and implications for estimating transmission. PLOS Neglected Tropical Diseases. 2018 Jan 22;12(1):e0006161.

69. Dalziel BD, Chamberlain SD, Ariza CA, Daitch AL, Philips PP, Singh I. Long-range local influenza forecasts via distributed syndromic monitoring: preliminary results. medRxiv. 2020 Jun 9;2020.06.07.20078956.

70. Dana S, Simas AB, Filardi BA, Rodriguez RN, Valiengo L da CL, Gallucci-Neto J. Brazilian Modeling of COVID-19(BRAM-COD): a Bayesian Monte Carlo approach for COVID-19 spread in a limited data set context. medRxiv. 2020 May 17;2020.04.29.20081174.

71. Dharmaratne S, Sudaraka S, Abeyagunawardena I, Manchanayake K, Kothalawala M, Gunathunga W. Estimation of the basic reproduction number (R0) for the novel coronavirus disease in Sri Lanka. Virology Journal. 2020 Oct 7;17(1):144.

72. Dighe A, Cattarino L, Cuomo-Dannenburg G, Skarp J, Imai N, Bhatia S, et al. Response to COVID-19 in South Korea and implications for lifting stringent interventions. BMC Medicine. 2020 Oct 9;18(1):321.

73. Diniz-Filho JAF, Jardim L, Toscano CM, Rangel TF. The effective reproductive number (Rt) of COVID-19 and its relationship with social distancing. medRxiv. 2020 Dec 29;2020.07.28.20163493.

74. Durmuş H, Gökler ME, Metintaş S. The Effectiveness of Community-based Social Distancing for Mitigating the Spread of the COVID-19 Pandemic in Turkey. J Prev Med Public Health. 2020 Nov;53(6):397–404.

75. Ewing A, Lee EC, Viboud C, Bansal S. Contact, Travel, and Transmission: The Impact of Winter Holidays on Influenza Dynamics in the United States. J Infect Dis. 2017 Mar 1;215(5):732–9.

76. Felix FHC, Fontenele J. Instantaneous R calculation for COVID-19 epidemic in Brazil. medRxiv. 2020 Apr 29;2020.04.23.20077172.

77. Ferreira DS de A, Lima FM de, Freitas JR de, Nascimento GILA, Alves DAN da S, Gomes DA, et al. Transitivity of COVID-19, based on parameters R_0 and R_((t)) in Pombos/PE municipality, Brazil. RSD. 2020 Sep 20;9(10):e1139108441–e1139108441.

78. Força-Tarefa de Modelagem da COVID-19. Análise do efeito das medidas de contenção à propagação da COVID-19 em Belo Horizonte (23/03 a 29/03) [Internet]. Federal University of Minas Gerais; 2020 Apr [cited 2021 Jan 11]. Available from: https://ufmg.br/storage/2/5/a/7/25a7163c7fb5575ab6d81b5a05bfd844_15863100172762_298779967.pdf

79. Forsyth O. Uncertainty and lockdown in COVID-19: An incomplete information SIR model. Covid Economics. 2020 Oct 15;(52):1–38.

80. Franco D, Gonzalez C, Abrego LE, Carrera J-P, Diaz Y, Caicedo Y, et al. Early Transmission Dynamics, Spread, and Genomic Characterization of SARS-CoV-2 in Panama. Emerg Infect Dis. 2021 Feb;27(2):612–5.

81. Frausto-Martínez O, Aguilar-Becerra CD, Colín-Olivares O, Sánchez-Rivera G, Hafsi A, Contreras-Tax AF, et al. COVID-19, Storms, and Floods: Impacts of Tropical Storm Cristobal in the Western Sector of the Yucatan Peninsula, Mexico. Sustainability. 2020 Jan;12(23):9925.

82. Freitas JR de, Ferreira DS de A, Lima FM de, Nascimento GILA, Alves DAN da S, Gomes DA, et al. Estimativa do número efetivo de reprodução de SARS-CoV-2 em Vitória de Santo Antão/PE, Brasil. RSD. 2020 Sep 7;9(9):e794997922–e794997922.

83. Fung IC-H, Hung YW, Ofori SK, Muniz-Rodriguez K, Lai P-Y, Chowell G. SARS-CoV-2 Transmission in Alberta, British Columbia, and Ontario, Canada, January 1-July 6, 2020. medRxiv. 2020 Aug 26;2020.07.18.20156992.

84. Galbán-García E, Más-Bermejo P. COVID-19 in Cuba: Assessing the National Response. MEDICC Review. 2020 Oct 10;22(4):29–34.

85. Gao S, Rao J, Kang Y, Liang Y, Kruse J, Dopfer D, et al. Association of Mobile Phone Location Data Indications of Travel and Stay-at-Home Mandates With COVID-19 Infection Rates in the US. JAMA Netw Open. 2020 Sep 8;3(9):e2020485.

86. Gastañaduy PA, Paul P, Fiebelkorn AP, Redd SB, Lopman BA, Gambhir M, et al. Assessment of the Status of Measles Elimination in the United States, 2001–2014. Am J Epidemiol. 2017 Apr 1;185(7):562–9.

87. Gastañaduy PA, Funk S, Paul P, Tatham L, Fisher N, Budd J, et al. Impact of Public Health Responses During a Measles Outbreak in an Amish Community in Ohio: Modeling the Dynamics of Transmission. Am J Epidemiol. 2018 Sep;187(9):2002–10.

88. Gastañaduy PA, Funk S, Lopman BA, Rota PA, Gambhir M, Grenfell B, et al. Factors Associated With Measles Transmission in the United States During the Postelimination Era. JAMA Pediatr. 2020 Jan 1;174(1):56.

89. Ghose A, Bhattacharya S, Karthikeyan AS, Kudale A, Monteiro JM, Joshi A, et al. Community prevalence of antibodies to SARS-CoV-2 and correlates of protective immunity in an Indian metropolitan city. medRxiv. 2020 Nov 30;2020.11.17.20228155.

90. Governo do Estado do Rio Grande do Norte Secretaria de Estado da Saúde Pública. Recomendações do comitê de especialistas da SESAP-RN para o enfrentamento da pandemia pela COVID-19 [Recommendation No. 023/2021] [Internet]. [cited 2021 Jun 4]. Available from: https://portalcovid19.saude.rn.gov.br/wp-content/uploads/2020/05/recomendacoes-comite-23.pdf

91. Gozzi N, Tizzoni M, Chinazzi M, Ferres L, Vespignani A, Perra N. Estimating the effect of social inequalities in the mitigation of COVID-19 across communities in Santiago de Chile. medRxiv. 2020 Oct 13;2020.10.08.20204750.

92. Grice P, Grice S, Laugesen RS. Calculating the Effective Reproduction Number for COVID-19 Using a New Process for Various Countries [Internet]. CloseAssociate and University of Illinois at Urbana-Champaign; 2020 May p. 19. Available from: https://e2a6b380-4bd5-43a8-b33f-68e981870108.usrfiles.com/ugd/e2a6b3_093ee4ee50fc442b92c29614902487c1.pdf

93. Guevara A. Mathetical modeling COVID 19 Loja province. Pre-print [Internet]. 2020 Apr 22 [cited 2021 Jan 30]; Available from: https://www.researchgate.net/publication/340846424_Mathetical_modeling_COVID_19_Loja_province

94. Gupta M, Mohanta SS, Rao A, Parameswaran GG, Agarwal M, Arora M, et al. Transmission dynamics of the COVID-19 epidemic in India and modeling optimal lockdown exit strategies. International Journal of Infectious Diseases. 2021 Feb 1;103:579–89.

95. Guzzetta G, Riccardo F, Marziano V, Poletti P, Trentini F, Bella A, et al. Impact of a Nationwide Lockdown on SARS-CoV-2 Transmissibility, Italy - Volume 27, Number 1—January 2021 - Emerging Infectious Diseases journal - CDC. Emerging Infectious Diseases. 27(1):267–70.

96. Hart WS, Maini PK, Thompson RN. High infectiousness immediately before COVID-19 symptom onset highlights the importance of contact tracing. medRxiv. 2020 Nov 23;2020.11.20.20235754.

97. Hasan A, Susanto H, Tjahjono V, Kusdiantara R, Putri E, Hadisoemarto P, et al. A new estimation method for COVID-19 time-varying reproduction number using active cases. medRxiv. 2021 Jan 30;2020.06.28.20142158.

98. Haug N, Geyrhofer L, Londei A, Dervic E, Desvars-Larrive A, Loreto V, et al. Ranking the effectiveness of worldwide COVID-19 government interventions. Nat Hum Behav. 2020 Dec;4(12):1303–12.

99. Hotz T, Glock M, Heyder S, Semper S, Böhle A, Krämer A. Monitoring the spread of COVID-19 by estimating reproduction numbers over time. arXiv:200408557 [q-bio, stat] [Internet]. 2020 Apr 18 [cited 2021 Jun 4]; Available from: http://arxiv.org/abs/2004.08557

100. Hu F-C. The Estimated Time-Varying Reproduction Numbers during the Ongoing Pandemic of the Coronavirus Disease 2019 (COVID-19) in 12 Selected Countries outside China. medRxiv. 2020 May 14;2020.05.10.20097154.

101. Hukić M, Ponjavić M. COVID-19 pandemic in Bosnia and Herzegovina: March – June 2020 [Internet]. Academy of Sciences and Arts of Bosnia and Herzegovina; 2020 [cited 2021 Jun 4]. Available from: https://publications.anubih.ba/handle/123456789/710

102. Hukic M, Ponjavic M, Tahirovic E, Karabegovic A, Ferhatbegovic E, Travar M, et al. SARS-CoV-2 virus outbreak and the emergency public health measures in Bosnia and Herzegovina: January – July 2020. Bosn J Basic Med Sci. 2021 Feb;21(1):111–6.

103. International Ebola Response Team, Agua-Agum J, Ariyarajah A, Aylward B, Bawo L, Bilivogui P, et al. Exposure Patterns Driving Ebola Transmission in West Africa: A Retrospective Observational Study. PLoS Med [Internet]. 2016 Nov 15 [cited 2021 Jun 4];13(11). Available from: https://www.ncbi.nlm.nih.gov/pmc/articles/PMC5112802/

104. Jombart T, Cori A, Didelot X, Cauchemez S, Fraser C, Ferguson N. Bayesian Reconstruction of Disease Outbreaks by Combining Epidemiologic and Genomic Data. PLOS Computational Biology. 2014 Jan 23;10(1):e1003457.

105. Jordan A, Sadler RJ, Sawford K, Andel M van, Ward M, Cowled B. Mycoplasma bovis outbreak in New Zealand cattle: An assessment of transmission trends using surveillance data. Transboundary and Emerging Diseases [Internet]. [cited 2021 Jun 4];n/a(n/a). Available from: https://onlinelibrary.wiley.com/doi/abs/10.1111/tbed.13941

106. Kajitani Y, Hatayama M. Explaining the Effective Reproduction Number of COVID-19 through Mobility and Enterprise Statistics: Evidence from the First Wave in Japan. medRxiv. 2020 Dec 4;2020.10.08.20209643.

107. Kendall M, Milsom L, Abeler-Dörner L, Wymant C, Ferretti L, Briers M, et al. Epidemiological changes on the Isle of Wight after the launch of the NHS Test and Trace programme: a preliminary analysis. The Lancet Digital Health. 2020 Dec 1;2(12):e658–66.

108. Kirsch TD, Moseson H, Massaquoi M, Nyenswah TG, Goodermote R, Rodriguez-Barraquer I, et al. Impact of interventions and the incidence of ebola virus disease in Liberia—implications for future epidemics. Health Policy Plan. 2017 Mar;32(2):205–14.

109. Koh WC, Naing L, Wong J. Estimating the impact of physical distancing measures in containing COVID-19: an empirical analysis. International Journal of Infectious Diseases. 2020 Nov 1;100:42–9.

110. Kraemer MUG, Pigott DM, Hill SC, Vanderslott S, Reiner RC, Stasse S, et al. Dynamics of conflict during the Ebola outbreak in the Democratic Republic of the Congo 2018–2019. BMC Medicine. 2020 Apr 27;18(1):113.

111. Kumar K, Meitei WB, Singh A. Projecting the future trajectory of COVID-19 infections in India using the susceptible-infected-recovered (SIR) model [Internet]. International Institute for Population Sciences [Ministry of Health & Family Welfare, Government of India]; 2020 May. (IIPS Analytical Series on COVID 19). Report No.: 7. Available from: https://www.iipsindia.ac.in/sites/default/files/iips_covid19_pfti.pdf

112. Lai CKC, Ng RWY, Wong MCS, Chong KC, Yeoh YK, Chen Z, et al. Epidemiological characteristics of the first 100 cases of coronavirus disease 2019 (COVID-19) in Hong Kong Special Administrative Region, China, a city with a stringent containment policy. International Journal of Epidemiology. 2020 Aug 1;49(4):1096–105.

113. Laxminarayan R, Wahl B, Dudala SR, Gopal K, B CM, Neelima S, et al. Epidemiology and transmission dynamics of COVID-19 in two Indian states. Science. 2020 Nov 6;370(6517):691–7.

114. Lee J, Jung E. A spatial–temporal transmission model and early intervention policies of 2009 A/H1N1 influenza in South Korea. Journal of Theoretical Biology. 2015 Sep 7;380:60–73.

115. Lee W, Hwang S-S, Song I, Park C, Kim H, Song I-K, et al. COVID-19 in South Korea: epidemiological and spatiotemporal patterns of the spread and the role of aggressive diagnostic tests in the early phase. International Journal of Epidemiology. 2020 Aug 1;49(4):1106–16.

116. Leung K, Wu JT, Liu D, Leung GM. First-wave COVID-19 transmissibility and severity in China outside Hubei after control measures, and second-wave scenario planning: a modelling impact assessment. The Lancet. 2020 Apr 25;395(10233):1382–93.

117. Leung K, Wu JT, Xu K, Wein LM. No Detectable Surge in SARS-CoV-2 Transmission Attributable to the April 7, 2020 Wisconsin Election. Am J Public Health. 2020 Aug;110(8):1169–70.

118. Leung K, Wu JT, Leung GM. Real-time tracking and prediction of COVID-19 infection using digital proxies of population mobility and mixing. Nat Commun. 2021 Mar 8;12(1):1501.

119. Lipsitch M, Joshi K, Cobey SE. Comment on Pan A, Liu L, Wang C, et al., 'Association of Public Health Interventions With the Epidemiology of the COVID-19 Outbreak in Wuhan, China,' JAMA, Published online April 10, 2020, doi:10.1001/jama.2020.6130. 2020 Apr 21 [cited 2021 Jun 4]; Available from: https://dash.harvard.edu/handle/1/42660128

120. Liu Q-H, Bento AI, Yang K, Zhang H, Yang X, Merler S, et al. The COVID-19 outbreak in Sichuan, China: Epidemiology and impact of interventions. PLOS Computational Biology. 2020 Dec 28;16(12):e1008467.

121. Liu T, Hu J, Xiao J, He G, Kang M, Rong Z, et al. Time-varying transmission dynamics of Novel Coronavirus Pneumonia in China. bioRxiv. 2020 Feb 13;2020.01.25.919787.

122. Liu W, Wang W, Hua S, Xie C, Wang B, Qiu W. Geographic information system methods tell spatiotemporal transmission and its drivers of COVID-19 in Wuhan, China at street-level. Pre-print [Internet]. 2020 Nov 11 [cited 2021 Jun 4]; Available from: https://www.researchsquare.com/article/rs-104986/v1

123. Liu X, Xu X, Li G, Xu X, Sun Y, Wang F, et al. Differential impact of non-pharmaceutical public health interventions on COVID-19 epidemics in the United States. Pre-print [Internet]. 2020 Dec 28 [cited 2021 Jun 4]; Available from: https://www.researchsquare.com/article/rs-60056/v3

124. Liu Z, Yin H, Kammen D. The Interaction between Population Age Structure and Policy Interventions on the Spread of COVID-19. Pre-print [Internet]. 2020 Aug 24 [cited 2021 Jun 4]; Available from: https://www.researchsquare.com/article/rs-57858/v1

125. Manrique-Abril FG, Téllez-Piñerez C, Pacheco-López M. Estimation of time-varying reproduction numbers of COVID-19 in American countries with regards to non-pharmacological interventions. F1000Res. 2020 Jul 31;9:868.

126. Martínez VP, Paola ND, Alonso DO, Pérez-Sautu U, Bellomo CM, Iglesias AA, et al. “Super-Spreaders” and Person-to-Person Transmission of Andes Virus in Argentina. New England Journal of Medicine [Internet]. 2020 Dec 2 [cited 2021 Jun 4]; Available from: https://www.nejm.org/doi/10.1056/NEJMoa2009040

127. Masoud M, Gewaifel G, Gamaleldin N. Transmissibility and mortality trends of COVID-19 epidemic in Egypt. Alexandria Journal of Medicine. 2020 Jan 1;56(1):189–95.

128. Menéndez J. A poor-man’s approach to the effective reproduction number: the COVID-19 case. medRxiv. 2020 Apr 27;2020.04.22.20076430.

129. Miller AC, Hannah L, Futoma J, Foti NJ, Fox EB, D’Amour A, et al. Statistical deconvolution for inference of infection time series. medRxiv. 2020 Oct 20;2020.10.16.20212753.

130. Miramontes O. Entendamos el COVID-19 en México [Internet]. Universidad Nacional Autónoma de México; 2020 Sep p. 37. Available from: http://scifunam.fisica.unam.mx/mir/corona19/covid19.pdf

131. Moirano G, Richiardi L, Novara C, Maule M. Approaches to Daily Monitoring of the SARS-CoV-2 Outbreak in Northern Italy. Front Public Health [Internet]. 2020 [cited 2021 Jun 4];8. Available from: https://www.frontiersin.org/articles/10.3389/fpubh.2020.00222/full

132. Moirano G, Schmid M, Barone-Adesi F. Short-Term Effects of Mitigation Measures for the Containment of the COVID-19 Outbreak: An Experience From Northern Italy. Disaster Med Public Health Prep. :1–2.

133. Moon S-G, Kim Y-K, Son W-S, Kim J-H, Choi J, Na B-J, et al. Time-variant reproductive number of COVID-19 in Seoul, Korea. Epidemiol Health [Internet]. 2020 Jun 28 [cited 2021 Jun 4];42. Available from: https://www.ncbi.nlm.nih.gov/pmc/articles/PMC7644928/

134. Mora X. El nombre de reproducció de la COVID-19 i el model SIR. L’efecte dels retards de comptabilització [Catalan]. Materials matemàtics. 2020 May 25;2020(2):19.

135. Moreno P, Moratorio G, Iraola G, Fajardo Á, Aldunate F, Pereira-Gómez M, et al. An effective COVID-19 response in South America: the Uruguayan Conundrum. medRxiv. 2020 Jul 27;2020.07.24.20161802.

136. Morley CP, Anderson KB, Shaw J, Stewart T, Thomas SJ, Wang D. Social Distancing Metrics and Estimates of SARS-CoV-2 Transmission Rates: Associations Between Mobile Telephone Data Tracking and R. Journal of Public Health Management and Practice. 2020 Dec;26(6):606–12.

137. Muniz-Rodriguez K, Chowell G, Schwind JS, Ford R, Ofori SK, Ogwara CA, et al. Time-varying reproduction numbers of COVID-19 in Georgia, USA, March 2-June 14, 2020. medRxiv. 2020 Jul 28;2020.05.19.20107219.

138. Musa SS, Zhao S, Wang MH, Habib AG, Mustapha UT, He D. Estimation of exponential growth rate and basic reproduction number of the coronavirus disease 2019 (COVID-19) in Africa. Infectious Diseases of Poverty. 2020 Jul 16;9(1):96.

139. Najafi F, Izadi N, Hashemi-Nazari S-S, Khosravi-Shadmani F, Nikbakht R, Shakiba E. Serial interval and time-varying reproduction number estimation for COVID-19 in western Iran. New Microbes New Infect [Internet]. 2020 Jun 14 [cited 2021 Jun 4];36. Available from: https://www.ncbi.nlm.nih.gov/pmc/articles/PMC7293842/

140. Nguyen HG, Nguyen TV. An epidemiologic profile of COVID-19 patients in Vietnam. medRxiv. 2020 Apr 15;2020.04.10.20061226.

141. O'Driscoll M, Harry C, Donnelly CA, Cori A, Dorigatti I. A Comparative Analysis of Statistical Methods to Estimate the Reproduction Number in Emerging Epidemics, With Implications for the Current Coronavirus Disease 2019 (COVID-19) Pandemic. Clinical Infectious Diseases [Internet]. 2020 Oct 20 [cited 2021 Jun 4];(ciaa1599). Available from: https://doi.org/10.1093/cid/ciaa1599

142. Olson MF, Ndeffo-Mbah ML, Juarez JG, Garcia-Luna S, Martin E, Borucki MK, et al. High Rate of Non-Human Feeding by Aedes aegypti Reduces Zika Virus Transmission in South Texas. Viruses [Internet]. 2020 Apr 17 [cited 2021 Jun 4];12(4). Available from: https://www.ncbi.nlm.nih.gov/pmc/articles/PMC7232486/

143. Ontario Agency for Health Protection and Promotion (Public Health Ontario). Enhanced Epidemiological Summary: COVID-19 Regional Incidence and Time to Case Notification in Ontario [Internet]. Ontario Agency for Health Protection and Promotion: Public Health Ontario; p. 181. Available from: https://www.publichealthontario.ca/-/media/documents/ncov/epi/covid-19-regional-epi-summary-report.pdf?la=en

144. Otomaru H, Kamigaki T, Tamaki R, Okamoto M, Alday PP, Tan AG, et al. Transmission of Respiratory Syncytial Virus Among Children Under 5 Years in Households of Rural Communities, the Philippines. Open Forum Infect Dis [Internet]. 2019 Mar 11 [cited 2021 Jun 4];6(3). Available from: https://www.ncbi.nlm.nih.gov/pmc/articles/PMC6411217/

145. Oyinlola MA, Osayomi T, Adeniyi O. Empirical Modelling of Confirmed COVID-19 Cases in Nigeria: Forecasts and Implications. Social Sciences & Humanities Open [Pre-print]. 2020 May 12;16.

146. Páez GN, Cerón JF, Cortés S, Quiroz AJ, Zea JF, Franco C, et al. Alternativas en la estimación del número básico de reproducción de una enfermedad [Internet]. Sociedad Colombiana de Matemáticas; [cited 2021 Jan 30] p. 23. Available from: http://scm.org.co/wp-content/uploads/2020/09/Paez-et-al.-Alternativas-en-estimaci%C3%B3n-del-n%C3%BAmero-de-reproducci%C3%B3n-.pdf

147. Pan A, Liu L, Wang C, Guo H, Hao X, Wang Q, et al. Association of Public Health Interventions With the Epidemiology of the COVID-19 Outbreak in Wuhan, China. JAMA. 2020 May 19;323(19):1915.

148. Park SH, Kim WJ, Yoo J-H, Choi J-H. Epidemiologic Parameters of the Middle East Respiratory Syndrome Outbreak in Korea, 2015. Infect Chemother. 2016 Jun;48(2):108–17.

149. Park SW, Sun K, Viboud C, Grenfell BT, Dushoff J. Potential Role of Social Distancing in Mitigating Spread of Coronavirus Disease, South Korea. Emerg Infect Dis. 2020 Nov;26(11):2697–700.

150. Pasten H, Sepúlveda JC. El pedal acelerador de la epidemia [Internet]. Facultad de Matemáticas: Pontificia Universidad Católica de Chile; [cited 2021 Jan 26] p. 4. Available from: http://www.mat.uc.cl/~hector.pasten/preprints/Re.pdf

151. Paternina-Caicedo A, Choisy M, Garcia-Calavaro C, España G, Rojas-Suarez J, Dueñas C, et al. Social interventions can lower COVID-19 deaths in middle-income countries. medRxiv. 2020 Apr 23;2020.04.16.20063727.

152. Peng Z, Song W, Ding Z, Guan Q, Yang X, Xu Q, et al. Linking key intervention timings to rapid declining effective reproduction number to quantify lessons against COVID-19. Front Med. 2020 Jun 4;1–7.

153. Perkins TA, Rodriguez-Barraquer I, Manore C, Siraj AS, España G, Barker CM, et al. Heterogeneous local dynamics revealed by classification analysis of spatially disaggregated time series data. Epidemics. 2019 Dec 1;29:100357.

154. Perone CS. Analysis of the SARS-CoV-2 outbreak in Rio Grande do Sul / Brazil. arXiv:200710486 [physics, q-bio] [Internet]. 2020 Jul 26 [cited 2021 Jun 4]; Available from: http://arxiv.org/abs/2007.10486

155. Pitzer VE, Chitwood M, Havumaki J, Menzies NA, Perniciaro S, Warren JL, et al. The impact of changes in diagnostic testing practices on estimates of COVID-19 transmission in the United States. medRxiv [Internet]. 2020 Apr 24 [cited 2021 Jun 4]; Available from: https://www.ncbi.nlm.nih.gov/pmc/articles/PMC7276051/

156. Platteau J-P, Verardi V. How to exit Covid-19 lockdowns: Culture matters. Covid Economics. 2020 May 28;(23):1–57.

157. Pokhrel AK, Joshi YP, Bhattarai S. Epidemiological trend of COVID-19 in Nepal and the importance of social distancing to contain the virus. Applied Science and Technology Annals. 2020 Jun 30;1(1):15–25.

158. Pons-Salort M, Oberste MS, Pallansch MA, Abedi GR, Takahashi S, Grenfell BT, et al. The seasonality of nonpolio enteroviruses in the United States: Patterns and drivers. PNAS. 2018 Mar 20;115(12):3078–83.

159. Pulido D, Basurto D, Cándido M, Salas J. Geospatial Spread of the COVID-19 Pandemic in Mexico. arXiv:200607784 [physics, q-bio] [Internet]. 2020 Jun 13 [cited 2021 Jun 4]; Available from: http://arxiv.org/abs/2006.07784

160. Purkayastha S, Salvatore M, Mukherjee B. Are women leaders significantly better at controlling the contagion during the COVID-19 pandemic? J Health Soc Sci. 2020 Jun;5(2):231–40.

161. Ran J, Zhao S, Han L, Chong MKC, Qiu Y, Yang Y, et al. The changing patterns of COVID-19 transmissibility during the social unrest in the United States: A nationwide ecological study with a before-and-after comparison. One Health. 2021 Jun 1;12:100201.

162. Röst G, Bartha FA, Bogya N, Boldog P, Dénes A, Ferenci T, et al. Early Phase of the COVID-19 Outbreak in Hungary and Post-Lockdown Scenarios. Viruses. 2020 Jul;12(7):708.

163. Rubin D, Huang J, Fisher BT, Gasparrini A, Tam V, Song L, et al. Association of Social Distancing, Population Density, and Temperature With the Instantaneous Reproduction Number of SARS-CoV-2 in Counties Across the United States. JAMA Netw Open. 2020 Jul 23;3(7):e2016099.

164. Rypdal M, Rypdal V, Jakobsen PK, Ytterstad E, Løvsletten O, Klingenberg C, et al. Modelling suggests limited change in the reproduction number from reopening Norwegian kindergartens and schools during the COVID-19 pandemic. PLOS ONE. 2021 Feb 25;16(2):e0238268.

165. Ryu S, Ali ST, Jang C, Kim B, Cowling BJ. Effect of Nonpharmaceutical Interventions on Transmission of Severe Acute Respiratory Syndrome Coronavirus 2, South Korea, 2020 - Volume 26, Number 10—October 2020 - Emerging Infectious Diseases journal - CDC. Emerging Infectious Diseases [Internet]. 2020 Oct [cited 2021 Jun 4];26(10). Available from: https://wwwnc.cdc.gov/eid/article/26/10/20-1886_article

166. Ryu S, Noh E, Ali ST, Kim D, Lau EHY, Cowling BJ. Epidemiology and Control of Two Epidemic Waves of SARS-CoV-2 in South Korea. SSRN Electronic Journal [Pre-print] [Internet]. 2020 Oct 15 [cited 2021 Jun 4]; Available from: https://papers.ssrn.com/abstract=3684500

167. Sanchez L, Lorenzo-Luaces P, Sebrango C, Torres A, Fonte C, Crespo M, et al. How Mathematical Approaches Could Help Decision-Making to Epidemic Control? The Successful Experience against COVID-19 in Cuba. SSRN Electronic Journal [Pre-print] [Internet]. 2020 Jun 17 [cited 2021 Jun 4]; Available from: https://papers.ssrn.com/abstract=3629271

168. Sánchez Vargas HE, Ramos Sánchez LB, Galindo Llanes PÁ, Salgado Rodríguez A, Sánchez Vargas HE, Ramos Sánchez LB, et al. Modelación físico-matemática para la toma de decisiones frente a la COVID-19 en Cuba. Retos de la Dirección. 2020 Dec;14(2):54–85.

169. Santamaría L, Hortal J. COVID-19 effective reproduction number dropped during Spain's nationwide dropdown, then spiked at lower-incidence regions. Science of The Total Environment. 2021 Jan 10;751:142257.

170. Santana-Cibrian M, Acuña-Zegarra MA, Velasco-Hernandez JX, Santana-Cibrian M, Acuña-Zegarra MA, Velasco-Hernandez JX. Lifting mobility restrictions and the effect of superspreading events on the short-term dynamics of COVID-19. MBE. 2020;17(5):6240–58.

171. Sashittal P, Luo Y, Peng J, El-Kebir M. Characterization of SARS-CoV-2 viral diversity within and across hosts. bioRxiv. 2020 May 13;2020.05.07.083410.

172. Scire J, Nadeau S, Vaughan T, Brupbacher G, Fuchs S, Sommer J, et al. Reproductive number of the COVID-19 epidemic in Switzerland with a focus on the Cantons of Basel-Stadt and Basel-Landschaft. Swiss Medical Weekly [Internet]. 2020 May 4 [cited 2021 Jun 4];150(1920). Available from: https://smw.ch/article/doi/smw.2020.20271

173. Şenel K, Senel K, Özdinç M, Ozdinc M, Öztürkcan DS, Ozturkcan DS, et al. Instantaneous R for COVID-19 in Turkey: estimation by Bayesian statistical inference [Türkiye'de COVID-19 için anlık R hesaplaması: Bayesyen istatistiksel çıkarım ile tahmin]. Turkiye Klinikleri Journal of Medical Sciences. 2020 Jun 18;40(2):127–31.

174. Setti MO, Voutilainen A. Social Distancing with Movement Restrictions and the Effective Replication Number of COVID-19: Multi-Country Analysis Based on Phone Mobility Data. medRxiv. 2020 Oct 12;2020.10.08.20209064.

175. Severo D, Cruz GNF, de Araújo AC, Santos AM dos, Martins ALN, da Silva CF, et al. Nota Técnica dos Modelos Implementados pelo Coletivo Covid19br para Projeções de Cenários Futuros da Pandemia COVID-19 no Brasil. arXiv:200413488 [physics, q-bio] [Internet]. 2020 Apr 25 [cited 2021 Jun 4]; Available from: http://arxiv.org/abs/2004.13488

176. Shim E, Tariq A, Chowell G. Spatial variability in reproduction number and doubling time across two waves of the COVID-19 pandemic in South Korea, February to July, 2020. International Journal of Infectious Diseases. 2021 Jan 1;102:1–9.

177. Silva V, Paul N. Potential impact of earthquakes during the 2020 COVID-19 pandemic. Earthquake Spectra. 2021 Feb 1;37(1):73–94.

178. Singh BB, Lowerison M, Lewinson RT, Vallerand IA, Deardon R, Gill JPS, et al. Public health interventions slowed but did not halt the spread of COVID-19 in India. Transboundary and Emerging Diseases [Internet]. [cited 2021 Jun 4];n/a(n/a). Available from: https://onlinelibrary.wiley.com/doi/abs/10.1111/tbed.13868

179. Singh G, Patrikar S, Sarma PS, Soman B. Time-dependent dynamic transmission potential and instantaneous reproduction number of COVID-19 pandemic in India. medRxiv. 2020 Jul 16;2020.07.15.20154971.

180. Sirirungreung A, Yimchoho N, Monpungteim K, Pinthadis W, Jiraphongsa C. The 2009 Pandemic Influenza A Virus in an Outbreak during 2014 in Samut Prakan Province, Thailand | OSIR Journal. Outbreak, Surveillance and Investigation Reports. 2015 Sep;8(3):1–7.

181. Son H, Lee H, Lee M, Eun Y, Park K, Kim S, et al. Epidemiological characteristics of and containment measures for COVID-19 in Busan, Korea. Epidemiol Health [Internet]. 2020 Jun 1 [cited 2021 Jun 4];42. Available from: http://www.e-epih.org/journal/view.php?number=1102

182. Soucy J-PR, Sturrock SL, Berry I, Westwood DJ, Daneman N, MacFadden DR, et al. Estimating effects of physical distancing on the COVID-19 pandemic using an urban mobility index. medRxiv. 2020 May 24;2020.04.05.20054288.

183. Sousa WC de, Gonçalves DA, Cruz DB. COVID-19: Local/regional inequalities and impacts over critical healthcare infrastructure in Brazil. Ambient soc [Internet]. 2020 Jul 3 [cited 2021 Jun 3];23. Available from: https://www.scielo.br/j/asoc/a/jcQ8s5jTXVKNLFVYP8nQ5yt/?lang=en

184. Stockdale JE, Doig R, Min J, Mulberry N, Wang L, Elliott LT, et al. Long time frames to detect the impact of changing COVID-19 control measures. medRxiv. 2020 Jun 16;2020.06.14.20131177.

185. Talmoudi K, Safer M, Letaief H, Hchaichi A, Harizi C, Dhaouadi S, et al. Estimating transmission dynamics and serial interval of the first wave of COVID-19 infections under different control measures: a statistical analysis in Tunisia from February 29 to May 5, 2020. BMC Infectious Diseases. 2020 Dec 2;20(1):914.

186. Tamagusko T, Ferreira A. Data-Driven Approach to Understand the Mobility Patterns of the Portuguese Population during the COVID-19 Pandemic. Sustainability. 2020 Jan;12(22):9775.

187. Tang B, Xia F, Bragazzi NL, Wang X, He S, Sun X, et al. Lessons drawn from China and South Korea for managing COVID-19 epidemic: insights from a comparative modeling study. medRxiv. 2020 Mar 13;2020.03.09.20033464.

188. Tchole AIM, Li Z-W, Wei J-T, Ye R-Z, Wang W-J, Du W-Y, et al. Epidemic and control of COVID-19 in Niger: quantitative analyses in a least developed country. J Glob Health [Internet]. [cited 2021 Jun 4];10(2). Available from: https://www.ncbi.nlm.nih.gov/pmc/articles/PMC7719275/

189. Tebé C, Valls J, Satorra P, Tobías A. COVID19-world: a shiny application to perform comprehensive country-specific data visualization for SARS-CoV-2 epidemic. BMC Medical Research Methodology. 2020 Sep 21;20(1):235.

190. Thai PQ, Rabaa MA, Luong DH, Tan DQ, Quang TD, Quach H-L, et al. The First 100 Days of Severe Acute Respiratory Syndrome Coronavirus 2 (SARS-CoV-2) Control in Vietnam. Clinical Infectious Diseases. 2021 May 1;72(9):e334–42.

191. To T, Zhang K, Maguire B, Terebessy E, Fong I, Parikh S, et al. Correlation of ambient temperature and COVID-19 incidence in Canada. Sci Total Environ. 2021 Jan 1;750:141484.

192. Turasie AA. Temporal Dynamics in COVID-19 Transmission: Case of Some African Countries. Advances in Infectious Diseases. 2020 Apr 29;10(3):110–22.

193. Valcarcel B, Avilez JL, Torres-Roman JS, Poterico JA, Bazalar-Palacios J, Vecchia CL. The effect of early-stage public health policies in the transmission of COVID-19 for South American countries. Rev Panam Salud Publica [Internet]. 2020 Nov 20 [cited 2021 Jun 4];44. Available from: https://www.ncbi.nlm.nih.gov/pmc/articles/PMC7679045/

194. Valka F, Schuler C. Estimation and Interactive Visualization of the Time-Varying Reproduction Number Rt and the Time-Delay from Infection to Estimation. medRxiv. 2020 Sep 22;2020.09.19.20197970.

195. Valls J, Tobías A, Satorra P, Tebé C. COVID19-Tracker: una aplicación Shiny para analizar datos de la epidemia de SARS-CoV-2 en España. Gaceta Sanitaria. 2021 Jan 1;35(1):99–101.

196. Vaughan TG, Sciré J, Nadeau SA, Stadler T. Estimates of outbreak-specific SARS-CoV-2 epidemiological parameters from genomic data. medRxiv. 2020 Sep 14;2020.09.12.20193284.

197. Venkatesan P. National and state wise estimate of time varying reproduction number for COVID-19 in India during the nationwide lockdown. medRxiv. 2020 May 6;2020.05.01.20087197.

198. Villabona-Arenas CJ, Oliveira JL de, Sousa-Capra C de, Balarini K, Fonseca CRTP da, Zanotto PM de A. Epidemiological dynamics of an urban Dengue 4 outbreak in São Paulo, Brazil. PeerJ. 2016 Apr 5;4:e1892.

199. Villela D. How limitations in data of health surveillance impact decision making in the COVID-19 epidemic. SciELO Pre-prints [Internet]. 2020 Oct 9 [cited 2021 Jun 4]; Available from: https://preprints.scielo.org/index.php/scielo/preprint/view/1313/version/1407

200. Wang K, Zhao S, Li H, Song Y, Wang L, Wang MH, et al. Real-time estimation of the reproduction number of the novel coronavirus disease (COVID-19) in China in 2020 based on incidence data. Ann Transl Med [Internet]. 2020 Jun [cited 2021 Jun 4];8(11). Available from: https://www.ncbi.nlm.nih.gov/pmc/articles/PMC7327374/

201. Wang K, Gao J, Wang H, Wu X, Yuan Q, Cheng Y. Transmission potential and forecasting of the number of Coronavirus disease 2019 cases in Hubei Province, China. Research Square [Pre-print] [Internet]. 2020 Nov 23 [cited 2021 Jun 4]; Available from: https://www.researchsquare.com/article/rs-36755/v2

202. Wang Q, Zhao Y, Zhang Y, Qiu J, Li J, Yan N, et al. Could the ambient higher temperature decrease the transmissibility of COVID-19 in China? Environ Res. 2021 Feb;193:110576.

203. WHO Ebola Response Team. Ebola Virus Disease in West Africa — The First 9 Months of the Epidemic and Forward Projections. New England Journal of Medicine. 2014 Oct 16;371(16):1481–95.

204. Wong J, Chaw L, Koh WC, Alikhan MF, Jamaludin SA, Poh WWP, et al. Epidemiological Investigation of the First 135 COVID-19 Cases in Brunei: Implications for Surveillance, Control, and Travel Restrictions. Am J Trop Med Hyg. 2020 Oct;103(4):1608–13.

205. Wong MCS, Ng RWY, Chong KC, Lai CKC, Huang J, Chen Z, et al. Stringent containment measures without complete city lockdown to achieve low incidence and mortality across two waves of COVID-19 in Hong Kong. BMJ Global Health. 2020 Oct 1;5(10):e003573.

206. Worden L, Wannier R, Blumberg S, Ge AY, Rutherford GW, Porco TC. Estimation of effects of contact tracing and mask adoption on COVID-19 transmission in San Francisco: a modeling study. medRxiv [Internet]. 2020 Jun 11 [cited 2021 Jun 4]; Available from: https://www.ncbi.nlm.nih.gov/pmc/articles/PMC7302226/

207. Xia F, Xiao Y, Liu P, Cheke RA, Li X, Xia F, et al. Differences in how interventions coupled with effective reproduction numbers account for marked variations in COVID-19 epidemic outcomes. MBE. 2020;17(5):5085–98.

208. Xiao J, Hu J, He G, Liu T, Kang M, Rong Z, et al. The time-varying transmission dynamics of COVID-19 and synchronous public health interventions in China. Int J Infect Dis. 2021 Feb;103:617–23.

209. Xu J, Hussain S, Lu G, Zheng K, Wei S, Bao W, et al. Associations of Stay-at-Home Order and Face-Masking Recommendation with Trends in Daily New Cases and Deaths of Laboratory-Confirmed COVID-19 in the United States. Explor Res Hypothesis Med. 2020 Jul 8;1–10.

210. Yap FF, Yong M. Implementation of An Online COVID-19 Epidemic Calculator for Tracking the Spread of the Coronavirus in Singapore and Other Countries. medRxiv. 2020 Jun 4;2020.06.02.20120188.

211. Yu X. Impact of mitigating interventions and temperature on the instantaneous reproduction number in the COVID-19 pandemic among 30 US metropolitan areas. One Health. 2020 Dec 1;10:100160.

212. Yuan P, Li J, Aruffo E, Li Q, Zheng T, Ogden N, et al. Efficacy of “stay-at-home” policy and transmission of COVID-19 in Toronto, Canada: a mathematical modeling study. medRxiv. 2020 Oct 21;2020.10.19.20181057.

213. Zhang J, Litvinova M, Wang W, Wang Y, Deng X, Chen X, et al. Evolving epidemiology and transmission dynamics of coronavirus disease 2019 outside Hubei province, China: a descriptive and modelling study. The Lancet Infectious Diseases. 2020 Jul 1;20(7):793–802.

214. Zhang X, Pebody R, Charlett A, de Angelis D, Birrell P, Kang H, et al. Estimating and modelling the transmissibility of Middle East Respiratory Syndrome CoronaVirus during the 2015 outbreak in the Republic of Korea. Influenza Other Respir Viruses. 2017 Sep;11(5):434–44.

215. Zhang Y, Keegan LT, Qiu Y, Samore MH. The real time effective reproductive number for COVID-19 in the United States. medRxiv. 2020 May 13;2020.05.08.20095703.

216. Zhao H, Merchant NN, McNulty A, Radcliff TA, Cote MJ, Fischer R, et al. COVID-19: Short term prediction model using daily incidence data. medRxiv. 2020 Nov 24;2020.11.23.20237024.

217. Zhao L, Feng D, Ye R-Z, Wang H-T, Zhou Y-H, Wei J-T, et al. Outbreak of COVID-19 and SARS in mainland China: a comparative study based on national surveillance data. BMJ Open. 2020 Oct 1;10(10):e043411.

218. Zhao S, Musa SS, Qin J, He D. Associations between Public Awareness, Local Precipitation, and Cholera in Yemen in 2017. Am J Trop Med Hyg. 2019 Sep;101(3):521–4.

219. Zhao S, Musa SS, Qin J, He D. Phase-shifting of the transmissibility of macrolide-sensitive and resistant Mycoplasma pneumoniae epidemics in Hong Kong, from 2015 to 2018. International Journal of Infectious Diseases. 2019 Apr 1;81:251–3.

220. Zhao S, Liang X. A re-analysis to identify the structural breaks in COVID-19 transmissibility during the early phase of the outbreak in South Korea. International Journal of Infectious Diseases. 2020 Nov 1;100:10–1.

221. Zhao S, Tang X, Liang X, Chong MK, Ran J, Musa SS, et al. Modelling the Measles Outbreak at Hong Kong International Airport in 2019: A Data-Driven Analysis on the Effects of Timely Reporting and Public Awareness. IDR. 2020 Jun 17;13:1851–61.

222. Zhao S, Musa SS, Hebert JT, Cao P, Ran J, Meng J, et al. Modelling the effective reproduction number of vector-borne diseases: the yellow fever outbreak in Luanda, Angola 2015–2016 as an example. PeerJ [Internet]. 2020 Feb 27 [cited 2021 Jun 4];8. Available from: https://www.ncbi.nlm.nih.gov/pmc/articles/PMC7049463/

223. Zhao Y, Wang R, Li J, Zhang Y, Yang H, Zhao Y. Analysis of the Transmissibility Change of 2019-Novel Coronavirus Pneumonia and Its Potential Factors in China from 2019 to 2020. BioMed Research International. 2020 May 19;2020:e3842470.

224. Zheng L, Kang Q, Liao W, Chen X, Huang S, Liu D, et al. China's effective control and other countries' uncharted challenge against COVID-19: an epidemiological and modelling study. medRxiv. 2020 May 2;2020.04.28.20083899.

225. Zhu L, Liu X, Huang H, Avellán-Llaguno RD, Lazo MML, Gaggero A, et al. Meteorological impact on the COVID-19 pandemic: A study across eight severely affected regions in South America. Sci Total Environ. 2020 Nov 20;744:140881.

226. Zhuang Z, Zhao S, Lin Q, Cao P, Lou Y, Yang L, et al. Preliminary estimates of the reproduction number of the coronavirus disease (COVID-19) outbreak in Republic of Korea and Italy by 5 March 2020. International Journal of Infectious Diseases. 2020 Jun 1;95:308–10.

227. Djaafara BA, Imai N, Hamblion E, Impouma B, Donnelly CA, Cori A. A Quantitative Framework for Defining the End of an Infectious Disease Outbreak: Application to Ebola Virus Disease. American Journal of Epidemiology. 2021 Apr 6;190(4):642–51.

228. Djaafara BA, Whittaker C, Watson OJ, Verity R, Brazeau NF, Widyastuti W, et al. Quantifying the dynamics of COVID-19 burden and impact of interventions in Java, Indonesia. medRxiv. 2021 Feb 15;2020.10.02.20198663.

229. Esra R, Jamieson L, Fox MP, Letswalo D, Ngcobo N, Mngadi S, et al. Evaluating the impact of non-pharmaceutical interventions for SARS-CoV-2 on a global scale. medRxiv. 2020 Aug 5;2020.07.30.20164939.

230. Garcia LP, Traebert J, Boing AC, Santos GFZ, Pedebôs LA, d'Orsi E, et al. The potential spread of Covid-19 and government decision-making: a retrospective analysis in Florianópolis, Brazil. Rev bras epidemiol [Internet]. 2020 Sep 30 [cited 2021 Jun 4];23. Available from: https://www.scielo.br/j/rbepid/a/WJLGyZfwFkfPGVLMVW5y8ch/?lang=en

231. Huisman JS, Scire J, Angst DC, Neher RA, Bonhoeffer S, Stadler T. Estimation and worldwide monitoring of the effective reproductive number of SARS-CoV-2. medRxiv. 2020 Nov 30;2020.11.26.20239368.

232. Khosravi A, Chaman R, Rohani-Rasaf M, Zare F, Mehravaran S, Emamian MH. The basic reproduction number and prediction of the epidemic size of the novel coronavirus (COVID-19) in Shahroud, Iran. Epidemiology & Infection [Internet]. 2020 ed [cited 2021 Jun 4];148. Available from: https://www.cambridge.org/core/journals/epidemiology-and-infection/article/basic-reproduction-number-and-prediction-of-the-epidemic-size-of-the-novel-coronavirus-covid19-in-shahroud-iran/B9ED8A0969C0F36DCC72E4ABB45CCC19

233. Li C, Romagnani P, Anders H-J. Novel Criteria for When and How to Exit a COVID-19 Pandemic Lockdown. Front Big Data [Internet]. 2020 [cited 2021 Jun 4];3. Available from: https://www.frontiersin.org/articles/10.3389/fdata.2020.00026/full

234. Li Y, Undurraga EA, Zubizarreta JR. Effectiveness of Localized Lockdowns in the COVID-19 Pandemic. medRxiv. 2021 Mar 30;2020.08.25.20182071.

235. Parag KV. Improved estimation of time-varying reproduction numbers at low case incidence and between epidemic waves. medRxiv. 2021 May 19;2020.09.14.20194589.

236. Price DJ, Shearer FM, Meehan M, McBryde E, Golding N, McVernon J, et al. Estimating the case detection rate and temporal variation in transmission of COVID-19 in Australia: Technical Report 14th April 2020 [Internet]. Doherty Institute; 2020 Apr p. 11. Available from: https://www.doherty.edu.au/uploads/content_doc/Estimating_changes_in_the_transmission_of_COVID-19_April14-public-release.pdf

237. Salas J. Improving the Estimation of the COVID-19 Effective Reproduction Number using Nowcasting. arXiv:200709800 [physics, q-bio] [Internet]. 2021 Jan 25 [cited 2021 Jun 4]; Available from: http://arxiv.org/abs/2007.09800

238. Sherratt K, Abbott S, Meakin SR, Hellewell J, Munday JD, Bosse N, et al. Exploring surveillance data biases when estimating the reproduction number: with insights into subpopulation transmission of Covid-19 in England. medRxiv. 2021 Mar 18;2020.10.18.20214585.

239. Turbé H, Bjelogrlic M, Robert A, Gaudet-Blavignac C, Goldman J-P, Lovis C. Adaptive Time-Dependent Priors and Bayesian Inference to Evaluate SARS-CoV-2 Public Health Measures Validated on 31 Countries. Front Public Health [Internet]. 2021 [cited 2021 Jun 4];8. Available from: https://www.frontiersin.org/articles/10.3389/fpubh.2020.583401/full

240. Wang S, Yang X, Li L, Nadler P, Arcucci R, Huang Y, et al. A Bayesian Updating Scheme for Pandemics: Estimating the Infection Dynamics of COVID-19. IEEE Computational Intelligence Magazine. 2020 Nov;15(4):23–33.

241. Wilder B, Mina MJ, Tambe M. Tracking disease outbreaks from sparse data with Bayesian inference. arXiv:200905863 [cs, stat] [Internet]. 2020 Sep 12 [cited 2021 Jun 4]; Available from: http://arxiv.org/abs/2009.05863

242. Gostic KM, McGough L, Baskerville EB, Abbott S, Joshi K, Tedijanto C, et al. Practical considerations for measuring the effective reproductive number, Rt. PLOS Computational Biology. 2020 Dec 10;16(12):e1008409.

243. Parag K. kpzoo/EpiFilter [Internet]. 2021 [cited 2021 Jun 7]. Available from: https://github.com/kpzoo/EpiFilter

244. Tools to Make Developing R Packages Easier [Internet]. [cited 2022 Feb 24]. Available from: https://devtools.r-lib.org/

245. R Package Installation from Remote Repositories, Including GitHub [Internet]. [cited 2022 Feb 24]. Available from: https://remotes.r-lib.org/

246. JAGS - Just Another Gibbs Sampler [Internet]. [cited 2022 Feb 24]. Available from: https://mcmc-jags.sourceforge.io/

247. JAGS: Just Another Gibbs Sampler [Internet]. SourceForge. [cited 2022 Feb 24]. Available from: https://sourceforge.net/projects/mcmc-jags/

248. Stan [Internet]. stan-dev.github.io. [cited 2022 Feb 24]. Available from: //mc-stan.org/

249. RStan [Internet]. stan-dev.github.io. [cited 2022 Feb 24]. Available from: //mc-stan.org/users/interfaces/rstan

250. RStan Getting Started · stan-dev/rstan Wiki [Internet]. GitHub. [cited 2022 Feb 24]. Available from: https://github.com/stan-dev/rstan

251. Flu Pandemic [Internet]. [cited 2022 Mar 17]. Available from: https://imperialcollegelondon.github.io/epidemia/articles/flu.html

252. EpiEstim R package vignette. [Internet] [cited 2021 Oct 15] Available from: https://github.com/mrc-ide/EpiEstim/blob/master/vignettes/full_EpiEstim_vignette.Rmd
